# Characteristics and outcomes of acute kidney injury in hospitalized COVID-19 patients: A multicenter study by the Turkish society of nephrology

**DOI:** 10.1371/journal.pone.0256023

**Published:** 2021-08-10

**Authors:** Hakki Arikan, Savas Ozturk, Bulent Tokgoz, Belda Dursun, Nurhan Seyahi, Sinan Trabulus, Mahmud Islam, Yavuz Ayar, Numan Gorgulu, Serhat Karadag, Mahmut Gok, Esra Akcali, Feyza Bora, Zeki Aydın, Eda Altun, Elbis Ahbap, Mehmet Polat, Zeki Soypacacı, Ebru Gok Oguz, Sumeyra Koyuncu, Hulya Colak, İdris Sahin, Murside Esra Dolarslan, Ozant Helvacı, Ilhan Kurultak, Zehra Eren, Hamad Dheir, Melike Betul Ogutmen, Dilek Guven Taymez, Dilek Gibyeli Genek, Sultan Ozkurt, Elif Ari Bakır, Enver Yuksel, Tuncay Sahutoglu, Ozgur Akin Oto, Gulsah Boz, Erkan Sengul, Ekrem Kara, Serhan Tuglular

**Affiliations:** 1 Department of Internal Medicine, Division of Nephrology, Marmara University School of Medicine, Istanbul, Turkey; 2 Department of Nephrology, Haseki Training and Research Hospital, Istanbul, Turkey; 3 Department of Internal Medicine, Division of Nephrology, Erciyes University School of Medicine, Kayseri, Turkey; 4 Department of Internal Medicine, Division of Nephrology, Pamukkale University Medical School, Denizli, Turkey; 5 Department of Nephrology, Cerrahpasa Medical Faculty, Istanbul University, Istanbul, Turkey; 6 Division of Nephrology, Zonguldak Ataturk State Hospital, Zonguldak, Turkey; 7 Division of Nephrology, Bursa City Hospital, Faculty of Medicine, University of Health Sciences, Bursa, Turkey; 8 Department of Nephrology, Istanbul Bagcilar Training and Research Hospital, University of Health Sciences, Istanbul, Turkey; 9 Department of Nephrology, Sultan 2.Abdulhamid Han Training and Research Hospital, Istanbul, Turkey; 10 Department of Nephrology, Mersin University Faculty of Medicine, Mersin, Turkey; 11 Department of Internal Medicine, Division of Nephrology, Akdeniz University Faculty of Medicine, Antalya, Turkey; 12 Department of Nephrology, Kocaeli Darica Farabi Training and Research Hospital, University of Health Sciences, Kocaeli, Turkey; 13 Division of Nephrology, Golcuk Necati Celik State Hospital, Kocaeli, Turkey; 14 Department of Nephrology, Sisli Hamidiye Etfal Education and Research Hospital, Istanbul, Turkey; 15 Division of Nephrology, Nevsehir State Hospital, Nevsehir, Turkey; 16 Department of Nephrology, Ataturk Training and Research Hospital, University of Katip Celebi, Izmir, Turkey; 17 Department of Nephrology, Diskapi Yildirim Beyazit Education and Research Hospital, University of Health Sciences, Ankara, Turkey; 18 Division of Nephrology, Tepecik Education and Research Hospital University of Health Sciences, İzmir, Turkey; 19 Department of Internal Medicine, Division of Nephrology, Inonu University Faculty of Medicine, Malatya, Turkey; 20 Division of Nephrology, Trabzon Kanuni Education and Research Hospital, University of Health Sciences, Trabzon, Turkey; 21 Division of Nephrology, Yenimahalle Research and Training Hospital, Yildirim Beyazit University Faculty of Medicine, Ankara, Turkey; 22 Department of Nephrology, Trakya University Faculty of Medicine, Edirne, Turkey; 23 Department of Nephrology, Alanya Alaaddin Keykubat University School of Medicine, Antalya, Turkey; 24 Department of Internal Medicine, Division of Nephrology, Sakarya University Medical Faculty Education and Research Hospital, Sakarya, Turkey; 25 Division of Nephrology, Haydarpasa Numune Education and Research Hospital, University of Health Sciences, Istanbul, Turkey; 26 Nephrology and Dialysis Department, Kocaeli State Hospital, Kocaeli, Turkey; 27 Department of Nephrology, Faculty of Medicine, Mugla Sitki Kocman University, Mugla, Turkey; 28 Department of Nephrology, Faculty of Medicine, Eskisehir Osmangazi University, Eskisehir, Turkey; 29 Department of Nephrology, Bahcesehir University Hospital, Istanbul, Turkey; 30 Department of Nephrology, Gaziyasargil Training and Research Hospital, University of Health Sciences, Diyarbakir, Turkey; 31 Nephrology Unit, Sanliurfa Mehmet Akif Inan Training and Research Hospital, Sanliurfa, Turkey; 32 Department of Internal Medicine, Division of Nephrology, Istanbul Medical Faculty, Istanbul University, Istanbul, Turkey; 33 Division of Nephrology, Kayseri City Training and Research Hospital, Kayseri, Turkey; 34 Division of Nephrology, Kocaeli Derince Education and Research Hospital, University of Health Sciences, Kocaeli, Turkey; 35 Department of Internal Medicine, Division of Nephrology, Faculty of Medicine, Recep Tayyip Erdogan University, Rize, Turkey; Universita degli Studi di Perugia, ITALY

## Abstract

**Background:**

Acute kidney injury (AKI) is common in coronavirus disease-2019 (COVID-19) and the severity of AKI is linked to adverse outcomes. In this study, we investigated the factors associated with in-hospital outcomes among hospitalized patients with COVID-19 and AKI.

**Methods:**

In this multicenter retrospective observational study, we evaluated the characteristics and in-hospital renal and patient outcomes of 578 patients with confirmed COVID-19 and AKI. Data were collected from 34 hospitals in Turkey from March 11 to June 30, 2020. AKI definition and staging were based on the Kidney Disease Improving Global Outcomes criteria. Patients with end-stage kidney disease or with a kidney transplant were excluded. Renal outcomes were identified only in discharged patients.

**Results:**

The median age of the patients was 69 years, and 60.9% were males. The most frequent comorbid conditions were hypertension (70.5%), diabetes mellitus (43.8%), and chronic kidney disease (CKD) (37.6%). The proportions of AKI stages 1, 2, and 3 were 54.0%, 24.7%, and 21.3%, respectively. 291 patients (50.3%) were admitted to the intensive care unit. Renal improvement was complete in 81.7% and partial in 17.2% of the patients who were discharged. Renal outcomes were worse in patients with AKI stage 3 or baseline CKD. The overall in-hospital mortality in patients with AKI was 38.9%. In-hospital mortality rate was not different in patients with preexisting non-dialysis CKD compared to patients without CKD (34.4 versus 34.0%, p = 0.924). By multivariate Cox regression analysis, age (hazard ratio [HR] [95% confidence interval (95%CI)]: 1.01 [1.0–1.03], p = 0.035], male gender (HR [95%CI]: 1.47 [1.04–2.09], p = 0.029), diabetes mellitus (HR [95%CI]: 1.51 [1.06–2.17], p = 0.022) and cerebrovascular disease (HR [95%CI]: 1.82 [1.08–3.07], p = 0.023), serum lactate dehydrogenase (greater than two-fold increase) (HR [95%CI]: 1.55 [1.05–2.30], p = 0.027) and AKI stage 2 (HR [95%CI]: 1.98 [1.25–3.14], p = 0.003) and stage 3 (HR [95%CI]: 2.25 [1.44–3.51], p = 0.0001) were independent predictors of in-hospital mortality.

**Conclusions:**

Advanced-stage AKI is associated with extremely high mortality among hospitalized COVID-19 patients. Age, male gender, comorbidities, which are risk factors for mortality in patients with COVID-19 in the general population, are also related to in-hospital mortality in patients with AKI. However, preexisting non-dialysis CKD did not increase in-hospital mortality rate among AKI patients. Renal problems continue in a significant portion of the patients who were discharged.

## Introduction

As of December 2020, severe acute respiratory syndrome coronavirus 2 (SARS-CoV-2) affected more than 79 million individuals, causing 1.7 million deaths worldwide [1]. The kidneys are the second most frequently affected organ by SARS-CoV-2, after the lungs [2]. Although the initial reports from China reported that only 3–5% of coronavirus disease-2019 (COVID-19) cases developed acute kidney injury (AKI) [3–9], data from Europe and the United States (US) revealed an incidence of up to 34% [10–14].

AKI is an important complication of COVID-19 and is related to higher in-hospital mortality [15]. Furthermore, AKI is an important prognostic marker of disease severity and survival [15, 16]. The incidence of AKI is higher in patients who require intensive care support [5, 6, 15, 17–19] and 13.3–35.2% of patients with critical disease required kidney replacement therapy (KRT) [8, 10, 17, 19–21]. The severity of AKI was also related to the mortality rate, which was higher in those with AKI stage 2–3 or with high serum urea and creatinine levels at presentation [17], particularly in those who require KRT [16]. The mortality rate was increased approximately 1.73-fold (74.8%) in the intensive care unit (ICU) patients who received KRT [20]. In summary, AKI affects the prognosis of patients with COVID-19, increases the morbidity and mortality rates and the need for KRT, imposing a further burden on both patients and the healthcare system.

It is not clear whether AKI in patients with COVID-19 is causally related to a cytopathic effect of the virus or to the systemic inflammatory response and cytokine storm [22]. The pathogenetic mechanisms leading to AKI are complex and include prerenal AKI due to hypovolemia or cardiorenal syndrome and renal AKI secondary to cytokine release syndrome or rhabdomyolysis leading to acute tubular necrosis, virus-mediated injury, drug nephrotoxicity, and intravascular coagulation [23].

The impact of AKI on patient outcomes may differ due to factors such as geographical area, differences of health-care systems or hospital capacities. There is no large-scale study which document the outcomes of AKI in COVID-19 patients from geographical region in which Turkey is located. In addition, although the increased mortality risk in CKD patients with COVID 19 has been well demonstrated [24–27], it is not clear whether non-dialysis CKD has an impact on patient outcomes among COVID-19 patients with AKI. We were able to document the previous serum creatinine values in a significant proportion of the patients and thus could clearly demonstrate the presence of CKD in our study population. Therefore, we conducted a multicenter retrospective study to evaluate the demographic, and clinical findings and the patient and renal outcomes and related risk factors including non-dialysis-dependent CKD in hospitalized patients with COVID-19 and AKI in Turkey.

## Materials and methods

### Study design and participants

In this retrospective multicenter observational cohort study, we included hospitalized adult COVID-19 patients who had AKI at presentation or developed AKI during hospitalization in 34 centers in Turkey from March 11 to June 30, 2020. Data collection was terminated on July 13, 2020. This study was approved by the Scientific Committee of the Ministry of Health (approval no: 2020-05-04T14_16_17) and Marmara University Ethics Committee under a broad regulatory protocol allowing for analysis of patient-level data (Approval no: 09.2020.553). The Ethics Committee waived the requirement for informed consent.

Patients with end-stage kidney disease (ESKD), kidney transplantation, pregnancy and patients who were readmitted for AKI and/or any other reason were excluded. Patients who had been transferred to another hospital after hospitalization or not yet been discharged at the time the study end were excluded from the data analysis.

All patients with clinical and laboratory findings and chest computed tomography (CT) findings consistent with COVID-19 were included in the study, irrespective of the result of a reverse transcriptase-polymerase chain reaction (RT-PCR) assay for SARS-CoV-2 on a specimen obtained by nasopharyngeal swab.

**Fig 1:**
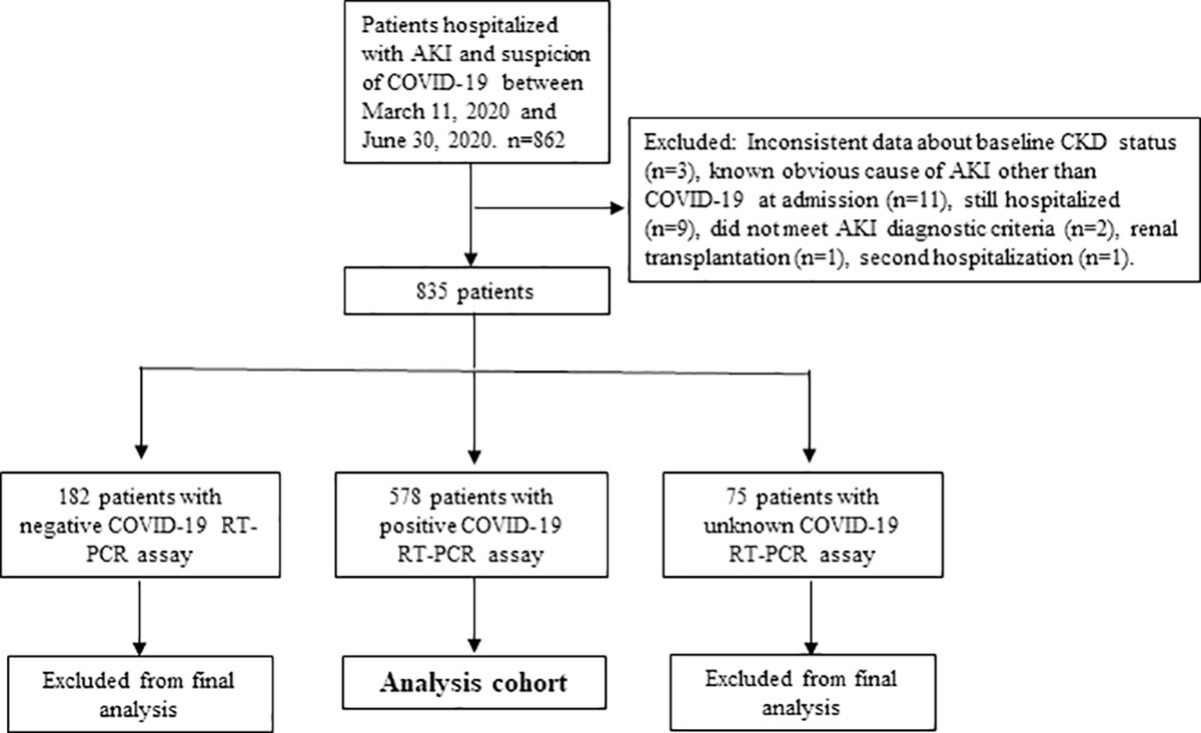
Flowchart of patient selection for the study. The diagram shows the numbers of individuals (*n*) excluded at different stages and the identification of cases for the analysis.

Among 862 patients, 18 patients were excluded from the study due to inconsistent data about baseline CKD status (n = 3), known obvious cause of AKI other than COVID-19 (n = 11), unmet AKI diagnostic criteria (n = 2), kidney transplantation (n = 1), second hospitalization (n = 2) (Fig 1). Among the remaining 835 patients, COVID-19 PCR was negative in 182 patients (21.8%), unknown in 75 (9%), and positive in 578 patients (69.2%). The demographic, laboratory, and clinical data of these patients according to AKI stage are shown in S1 Table. To prevent diagnostic bias, patients negative or unknown by RT-PCR assay and considered *possible* or *probable* cases according to the European Centers for Disease Control and Prevention (ECDC) criteria [28] were excluded from the final analysis and only COVID-19 PCR positive patients (confirmed cases) were used in the final analysis. Possible and probable cases were described as meeting the clinical criteria of COVID-19 including fever, cough or shortness of breath and the presence of the diagnostic imaging criteria, respectively. A flowchart of patient selection was shown in Fig 1.

### Data collection

We obtained demographic, clinical and laboratory data, radiologic findings, treatment characteristics and outcomes (patient and renal) from the electronic medical records. Previous comorbidities and medications were documented. Laboratory data included renal and liver function tests, albumin, electrolytes, lactate dehydrogenase (LDH), complete blood count, C-reactive protein (CRP), ferritin, procalcitonin, fibrinogen, d-dimer at the time of admission, as well as chest CT findings. Laboratory and clinical data during hospitalization [need for dialysis, admission to ICU, intubation, and extracorporeal membrane oxygenation (ECMO) requirement, macrophage activation syndrome (MAS), shock/severe hypotension, and secondary bacterial infection during hospitalization] were also recorded (Table 1 and S1 Table).

**Table 1:** Characteristics of COVID-19 RT-PCR positive patients during hospital admission and hospital stay, by AKI severity.

	Total (n = 578)	AKI 1 (n = 312)	AKI 2 (n = 143)	AKI 3, (n = 123)	p
**Age (years)**	69 (59–77)	67 (57–77)	67 (61–77)	72 (60–79)	0.169
**Male, n/N (%)**	352/578 (60.9)	184/312 (59)	85/143 (59.4)	83/123 (67.5)	0.241
**Comorbid conditions, n/N (%)**					
Diabetes mellitus	249/569 (43.8)	129/310 (41.6)	68/139 (48.9)	52/120 (43.3)	0.351
Hypertension	399/569 (70.5)	224/307 (73)	105/140 (75)	70/119 (58.8)	0.007
Chronic kidney disease	192/510 (37.6)	103/289 (35.6)	47/127 (37.0)	42/94 (44.7)	0.287
Obesity	27/462 (5.8)	12/250 (4.8)	8/121 (6.6)	7/91 (7.7)	0.552
Chronic obstructive pulmonary disease	89/549 (16.2)	50/301 (16.6)	19/133 (14.3)	20/115 (17.4)	0.772
Coronary heart disease	172/541 (31.8)	85/290 (29.3)	51/134 (38.1)	36/117 (30.8)	0.191
Heart failure	85/528 (16.1)	49/286 (17.1)	25/129 (19.4)	11/113 (9.7)	0.098
Cerebrovascular disease	39/546 (7.1)	18/298 (6)	10/135 (7.4)	11/113 (9.7)	0.426
Cancer	69/554 (12.5)	26/303 (8.6)	24/137 (17.5)	19/114 (16.7)	0.010
Chronic liver disease	10/556 (1.8)	3/304 (1)	4/137 (2.9)	3/115 (2.6)	0.338
Autoimmune/autoinflammatory disease	25/556 (4.5)	13/305 (4.3)	10/136 (7.4)	2/115 (1.7)	0.097
**Medications, n/N (%)**					
ACE-I or ARB	266/505 (52.7)	166/276 (60.1)	59/128 (46.1)	41/101 (40.6)	0.001
Ca antagonists	171/491 (34.8)	95/264 (36)	44/127 (34.6)	32/100 (32)	0.775
Beta blockers	216/491 (44)	115/262 (43.9)	62/130 (47.7)	39/99 (39.4)	0.455
Other antihypertensives	113/476 (23.7)	60/260 (23.1)	27/120 (22.5)	26/96 (27.1)	0.685
Insulin	122/484 (25.2)	58/259 (22.4)	38/123 (30.9)	26/102 (25.5)	0.202
Oral antidiabetics	127/486 (26.1)	71/260 (27.3)	31/123 (25.2)	25/103 (24.3)	0.808
Statins	88/477 (18.4)	41/254 (16.1)	27/122 (22.1)	20/101 (19.8)	0.346
Antiaggregant or anticoagulant drugs	251/501 (50.1)	133/265 (50.2)	67/130 (51.5)	51/106 (48.1)	0.871
**Smoking status, n/N (%)** 0.254					
Never	221/411 (53.8)	124/232 (53.4)	61/102 (59.8)	36/77 (46.8)	
Current	34/411 (8.3)	21/232 (9.1)	4/102 (3.9)	9/77 (11.7)	
Former	156/411 (38)	87/232 (37.5)	37/102 (36.3)	32/77 (41.6)	
**Symptoms related to COVID-19, n/N (%)**					
Fever	380/565 (67.3)	208/310 (67.1)	95/139 (68.3)	77/116 (66.4)	0.942
Fatigue	356/563 (63.2)	194/308 (63)	93/140 (66.4)	69/115 (60)	0.566
Dyspnea	375/569 (65.9)	185/310 (59.7)	100/140 (71.4)	90/119 (75.6)	0.002
Cough	408/564 (72.3)	225/310 (72.6)	101/139 (72.7)	82/115 (71.3)	0.962
Anorexia	132/540 (24.4)	86/301 (28.6)	29/129 (22.5)	17/110 (15.5)	0.020
Myalgia	192/542 (35.4)	107/303 (35.3)	46/131 (35.1)	39/108 (36.1)	0.985
Headache	82/545 (15)	47/300 (15.7)	16/133 (12)	19/112 (17)	0.507
Sore throat	72/546 (13.2)	39/303 (12.9)	22/133 (16.5)	11/110 (10)	0.315
Diarrhea	32/551 (5.8)	19/306 (6.2)	8/133 (6)	5/112 (4.5)	0.791
Others	40/508 (7.9)	22/282 (7.8)	9/120 (7.5)	9/106 (8.5)	0.960
Asymptomatic	4/519 (0.8)	3/283 (1.1)	1/127 (0.8)	0/109 (0)	
**Possible source of COVID-19, n/N (%)** 0.226					
Family-house	139/541 (25.7)	82/291 (28.2)	31/136 (22.8)	26/114 (22.8)	
Nursing home or prison	10/541 (1.8)	5/291 (1.7)	3/136 (2.2)	2/114 (1.8)	
Health institution	21/541 (3.9)	7/291 (2.4)	9/136 (6.6)	5/114 (4.4)	
Social life (meeting or dinner)	68/541 (12.6)	32/291 (11)	22/136 (16.2)	14/114 (12.3)	
Travel abroad	10/541 (1.8)	2/291 (0.7)	6/136 (4.4)	2/114 (1.8)	
Domestic travel	1/541 (0.2)	1/291 (0.3)	0/136 (0)	0/114 (0)	
Unknown	292/541 (54)	162/291 (55.7)	65/136 (47.8)	65/114 (57)	
**Radiologic examination, n/N (%)**					
Patients with a chest CT scan	563/572 (98.4)	304/309 (98.4)	141/143 (98.6)	118/120 (98.3)	1.000
Patients with specific chest CT findings	529/564 (93.8)	284/305 (93.1)	132/140 (94.3)	113/119 (95)	0.749
Patients with specific bilaterally chest CT findings	472/549 (86)	250/297 (84.2)	121/139 (87.1)	101/113 (89.4)	0.270
**Specific chest CT findings, n/N (%)**					
Ground glass opacity	503/535 (94)	270/289 (93.4)	129/137 (94.2)	104/109 (95.4)	0.755
Reticular opacity	155/28 (36.2)	84/232 (36.2)	36/110 (32.7)	35/86 (40.7)	0.515
Bronchial wall thickening	79/428 (18.5)	45/231 (19.5)	21/111 (18.9)	13/86 (15.1)	0.666
Pleural effusion	100/427 (23.4)	42/229 (18.3)	32/110 (29.1)	26/88 (29.5)	0.029
Thoracic lymphadenopathy	53/424 (12.5)	28/230 (12.2)	14/108 (13)	11/86 (12.8)	0.975
**Oxygen saturation in room air at diagnosis, n/N (%)** <0.001					
Normal	129/551 (23.4)	89/297 (30)	26/136 (19.1)	14/118 (11.9)	
90–95%	210/551 (38.1)	131/297 (44.1)	46/136 (33.8)	33/118 (28)	
<90%	212/551 (38.5)	77/297 (25.9)	64/136 (47.1)	71/118 (60.2)	
**Severity of COVID-19 infection, n/N (%)** <0.001					
Asymptomatic	13/578 (2.2)	9/312 (2.9)	3/143 (2.1)	1/123 (0.8)	
Mild to moderate	196/578 (33.9)	132/312 (42.3)	46/143 (32.2)	18/123 (14.6)	
Severe	256/578 (44.3)	132/312 (42.3)	62/143 (43.4)	62/123 (50.4)	
Critical	113/578 (19.6)	39/312 (12.5)	32/143 (22.4)	42/123 (34.1)	
**Time between first symptom and COVID-19 diagnosis (days)**	3 (2–5)	3 (2–5)	3 (2–5)	3 (3–4)	0.639
**Data for renal function within the last year**					
Serum creatinine (μmol/L)	86.6 (70.7–109.6)	88.4 (70.6–108.8)	84.9 (70.7–114.9)	85.7(70.7–114.9)	0.840
eGFR (mL/min/1.73 m^2^)	65.4 (40.6–96.6)	64.5 (41.8–95.3)	70.3 (39.1–100)	69.1 (40.2–100.4)	0.821
**CKD stages in patients with known eGFR within the last year, n/N (%)**			0.677		
eGFR>90 ml/min/1.73m2	130/447 (29.1)	66/239 (27.6)	35/115 (30.4)	29/93 (31.2)	
eGFR 60–90 ml/min/.73m^2^	126/447 (28.2)	71/239 (29.7)	33/115 (28.7)	22/93(23.7)	
CKD stage 3	113/447 (25.3)	60/239 (25.1)	27/115 (23.5)	26/93 (28)	
CKD stage 4	55/447 (12.3)	32/239 (13.4)	15/115 (13.0)	8/93(8.6)	
CKD stage 5	23/447 (5.1)	10/239 (4.2)	5/115 (4.3)	8/93 (8.6)	
**Laboratory parameters at hospital admission**					
Urea (mmol/L)	9.3 (6.0–14.0)	8.7 (5.7–12.3)	9.7 (6.6–16.1)	10.0 (6.8–18.3)	0.001
Creatinine (μmol/L)	117.6 (88.4–164.4)	114.9 (89.3–144.1)	123.8 (86.6–176.8)	123.8 (81.3–238.7)	0.118
Na (mmol/L)	137 (134–140)	137 (134–140)	136 (133–140)	136 (133–140)	0.625
K (mmol/L)	4.4 (3.9–4.8)	4.4 (4–4.7)	4.4 (3.9–4.8)	4.2 (3.9–4.8)	0.871
AST (U/L)	32 (20–49.4)	28 (19.2–42)	36 (20.5–57)	38.1 (24–61)	<0.001
ALT (U/L)	23 (14–35)	22 (14–33.1)	23 (15–37)	24 (14–44)	0.268
LDH (U/L)	320 (239–443)	296.5 (229.5–409.5)	321 (233–505)	381.5 (298–523)	<0.001
Albumin (g/L)	35 (30–39)	37 (32–39)	32 (28–37)	31 (28–36)	<0.001
Ferritin (μg/L)	360 (162–748)	290 (154–649)	376.5 (150–887)	502.5 (302.6–913)	<0.001
Fibrinogen (g/L)	4.7 (3.5–6.2)	4.6 (3.3–6.2)	4.5 (3.5–6.0)	5.0 (4.2–6.5)	0.140
D-dimer (mg/L)	14.6 (8.1–28.44)	13.2 (7.0–26.5)	15.8 (8.5–27.9)	15.1 (10.3–31.6)	0.218
Procalcitonin (ng/L)	350 (130–1145)	200 (100–760)	510 (270–1540)	840 (235–1700)	<0.001
Hemoglobin (g/dl)	12.1 (10.6–13.8)	12.7 (11–14.1)	11.7 (10.2–13.2)	11.6 (10.5–13)	<0.001
Leucocyte count(/mm3)	7835 (5500–11250)	7700 (5300–10630)	7870 (5540–11780)	8825 (5980–11600)	0.150
Neutrophil count (/mm3)	5750 (3700–9110)	5370 (3530–8100)	5750 (3700–9360)	7216 (4400–10600)	0.001
Lymphocyte count (/mm3)	1100 (700–1520)	1200 (800–1600)	1090 (750–1440)	805 (500–1390)	<0.001
Thrombocyte count (x1000/mm3)	202.5 (151–274)	211 (161–281)	187 (134–277)	197 (149–255)	0.041
CRP levels^†^, n/N (%) <0.001					
Normal	28/576 (4.9)	24/312 (7.7)	3/142 (2.1)	1/122 (0.8)	
1/5-fold x ULN	91/576 (15.8)	60/312 (19.2)	22/142 (15.5)	9/122 (7.4)	
5/10-fold x ULN	103/576 (17.9)	55/312 (17.6)	29/142 (20.4)	19/122 (15.6)	
10/20-fold x ULN	150/576 (26)	84/312 (26.9)	30/142 (21.1)	36/122 (29.5)	
>20-fold x ULN	204/576 (35.4)	89/312 (28.5)	58/142 (40.8)	57/122 (46.7)	
**Specific treatments for COVID-19, n/N (%)**					
Hydroxychloroquine	560/578 (96.9)	305/312 (97.8)	137/143 (95.8)	118/123 (95.9)	0.435
Oseltamivir	324/578 (56.1)	185/312 (59.3)	77/143 (53.8)	62/123 (50.4)	0.201
Macrolide	471/578 (81.5)	265/312 (84.9)	115/143 (80.4)	91/123 (74)	0.028
Favipiravir	369/578 (63.8)	180/312 (57.7)	99/143 (69.2)	90/123 (73.2)	0.003
Glucocorticoid	111/578 (19.2)	38/312 (12.2)	27/143 (18.9)	46/123 (37.4)	<0.001
Lopinavir-ritonavir	39/578 (6.7)	18/312 (5.8)	8/143 (5.6)	13/123 (10.6)	0.163
Tocilizumab	73/578 (12.6)	43/312 (13.8)	13/143 (9.1)	17/123 (13.8)	0.340
Convalescent plasma	19/578 (3.3)	6/312 (1.9)	4/143 (2.8)	9/123 (7.3)	0.019
Apheresis/immunoadsorption	9/578 (1.6)	2/312 (0.6)	1/143 (0.7)	6/123 (4.9)	0.006
IL-1 inhibitors (anakinra/canakinumab)	0/578 (0)	0/312 (0.3)	0/143 (0)	0/123 (0)	
JAK2 inhibitors	1/578 (0.2)	1/312 (0.3)	0/143 (0)	0/123 (0)	
**Unfavorable prognostic signs at any time during hospital stay, n/N (%)**					
Lymphopenia	614/834 (73.6)	303/437 (69.3)	169/217 (77.9)	142/180 (78.9)	0.013
Anemia (Hb <10 g/dL)	446/834 (53.5)	183/437 (41.9)	125/217 (57.6)	138/180 (76.7)	<0.001
Thrombocytopenia	261/830 (31.4)	119/437 (27.2)	71/215 (33)	71/178 (39.9)	0.008
LDH (>2-fold x ULN) ^‡^	400/811 (49.3)	173/431 (40.1)	109/209 (52.2)	118/171 (69)	<0.001
AST (>2-fold x ULN) ^‡‡^	350/831 (42.1)	138/436 (31.7)	103/217 (47.5)	109/178 (61.2)	<0.001
Macrophage activation syndrome	165/751 (22)	60/401 (15)	47/193 (24.4)	58/157 (36.9)	<0.001
Shock/severe hypotension	289/789 (36.6)	79/417 (18.9)	91/203 (44.8)	119/169 (70.4)	<0.001
Secondary bacterial infection	336/760 (44.2)	122/401 (30.4)	99/193 (51.3)	115/166 (69.3)	<0.001
CRP levels^†^			<0.001		
Normal	17/578 (2.9)	17/312 (5.4)	0/143 (0)	0/123 (0)	
1/5-fold x ULN	40/578 (6.9)	28/312 (9)	9/143 (6.3)	3/123 (2.4)	
5/10-fold x ULN	59/578 (10.2)	40/312 (12.8)	16/143 (11.2)	3/123 (2.4)	
10/20-fold x ULN	124/578 (21.5)	78/312 (25)	27/143 (18.9)	19/123 (15.4)	
>20-fold x ULN	338/578 (58.5)	149/312 (47.8)	91/143 (63.6)	98/123 (79.7)	
**Intensive care unit admission, n/N (%)**	291/578 (50.3)	100/312 (32.1)	83/143 (58)	108/123 (87.8)	<0.001
**Managements in the intensive care unit, n/N (%)**					
Intubation	232/288 (80.6)	66/100 (66)	65/81 (80.2)	101/107 (94.4)	<0.001
ECMO	18/265 (6.8)	5/92 (5.4)	1/72 (3.4)	12/101 (11.9)	0.021
Slow continuous dialysis	53/260 (20.4)	12/89 (13.5)	10/72 (13.9)	31/99 (31.3)	0.003
**Duration of stay in intensive care unit (days)**	9 (5–16)	8.5 (4–18)	8 (5–16)	9 (6–16)	0.851
**AKI timing, n/N (%)**					
AKI at hospital admission	251/578 (43.4)	145/312 (46.5)	53/143 (37.1)	53/123 (43.1)	0.170
AKI during hospital stay	327/578 (51.6)	167/132 (53.5)	90/143 (62.9)	70/123 (56.9)	0.170
**Suspected causes of AKI, n/N (%)** <0.001					
Prerenal	251/578 (43.4)	183/312 (58.7)	52/143 (36.4)	16/123 (13)	
Renal	311/578 (53.8)	119/312 (38.1)	91/143 (63.6)	101/123 (82.1)	
Postrenal	6/578 (1)	4/312 (1.3)	0/143 (0)	2/123 (1.6)	
Others	10/578 (1.7)	6/312 (1.9)	0/143 (0)	4/123 (3.3)	
**Suspected specific causes of AKI, n/N (%)** <0.001					
Dehydration	141/578 (24.4)	110/312 (35.3)	24/143 (16.8)	7/123 (5.7)	
GIS loss	10/578 (1.7)	7/312 (2.2)	2/143 (1.4)	1/123 (0.8)	
Heart failure	19/578 (3.3)	9/312 (2.9)	8/143 (5.6)	2/123 (1.6)	
Other prerenal causes	81/578 (14)	57/312 (18.3)	18/143 (12.6)	6/123 (4.9)	
Sepsis	233/578 (40.3)	80/312 (25.6)	63/143 (44.1)	90/123 (73.2)	
Thrombotic microangiopathy	8/578 (1.4)	3/312 (1)	2/143 (1.4)	3/123 (2.4)	
Extended prerenal causes	36/578 (6.2)	17/312 (5.4)	16/143 (11.2)	3/123 (2.4)	
Rhabdomyolysis	2/578 (0.3)	0/312 (0)	0/143 (0)	2/123 (1.6)	
Nephrotoxic drugs	32/578 (5.5)	19/312 (6.1)	10/143 (7)	3/123 (2.4)	
Postrenal (urological) causes	6/578 (1)	4/312 (1.3)	0/143 (0)	2/123 (1.6)	
Others	10/578 (1.7)	6/312 (1.9)	0/143 (0)	4/123 (3.3)	
**Time between hospitalization and AKI (days)**	5 (3–8)	4 (3–7)	5 (3–8)	5 (3–9)	0.304
**Dialysis requirement in the ward, n/N (%)**	91/554 (16.4)	11/304 (3.6)	19/134 (14.2)	61/116 (52.6)	<0.001
**KRT indications, n/N (%)**					
Increase in serum BUN/creatinine levels	42/115 (36,5)	9/19 (47,4)	9/27 (33,3)	24/69 (34,8)	
Hyperkalemia	10/115 (8.7)	0/19 (0)	3/27 (11.1)	7/69 (10.1)	
Metabolic acidosis	27/115 (23.5)	3/19 (15.8)	7/27 (25.9)	17/69 (24.6)	
Hypervolemia	22/115 (19.1)	3/19 (15.8)	4/27 (14.8)	15/69 (21.7)	
Severe uremic symptoms	3/115 (2.6)	1/19 (5.3)	0/27 (0)	2/69 (2.9)	
Others	11/115 (9.6)	3/19 (15.8)	4/27 (14.8)	4/69 (5.8)	
**Renal Outcome, n/N (%)** <0.001					
Complete recovery	285/349 (81.7)	213/251 (84.9)	59/76 (77.6)	13/22 (59.1)	
Partial recovery	60/349 (17.2)	37/251 (14.7)	16/76 (21.1)	7/22 (31.8)	
No improvement and/or dialysis dependence	4/349 (1.1)	1/251 (0.4)	1/76 (1.3)	2/22 (9.1)	
**Duration of AKI in discharged patients (days)**	6 (3–9)	5 (3–8)	6 (4–10)	9 (5–14)	<0.001
**Patient Outcome, n/N (%)**			<0.001		
Discharged	353/578 (61.1)	253/312 (81.1)	77/143 (53.8)	23/123 (18.7)	
Dead	225/578 (38.9)	59/312 (18.9)	66/143 (46.2)	100/123 (81.3)	
**Total hospital stays (days)**	12 (8–19)	12 (7.5–18)	13 (10–19)	14 (10–24)	0.014

ACE-I, angiotensin-converting enzyme inhibitors; ARB, angiotensin receptor blockers; COVID-19, coronavirus disease 2019; eGFR, estimated glomerular filtration rate; AST, aspartate aminotransferase; ALT, alanine aminotransferase; LDH, lactate dehydrogenase; x ULN, increase above upper normal limit; CRP, C-reactive protein; IL-1, interleukin 1; JAK2, Janus kinase; ECMO, extracorporeal membrane oxygenation; KRT, kidney replacement therapy.

Data were expressed as median [Q1-Q3] or as number (percent).

^†^The upper limit of the normal range of CRP was 5 mg/L (47.6 nmol/L).

^‡^The upper limit of the normal range of LDH was 248 U/L.

^‡‡^The upper limit of the normal range of AST was 37 U/L.

### Definitions and measurements

COVID-19 clinical status was classified as asymptomatic, mild-to-moderate, severe, or critical according to the World Health Organization Report of the WHO–China Joint Mission on Coronavirus Disease 2019 (*COVID-19*) [29].

The definition and staging of AKI were based on the 2012 Kidney Disease: Improving Global Outcomes (KDIGO) criteria [30]. As the KDIGO criteria may underestimate the incidence of AKI, we also used an automatically calculated prebuilt operational algorithm for AKI diagnosis and staging according to the serum creatinine value in the last 365 days [31].

Diagnosis and staging of AKI and CKD were performed according to a serum creatinine value within the previous 365 days. Patients who had no serum creatinine measurement in the last 365 days were not accepted as CKD, whereas those with renal function recovery during follow-up were considered as AKI. Renal recovery was defined as the serum creatinine level decreasing to the reference range and/or to >30% decrease compared to values at admission. Patients who experienced complete renal recovery at discharge were accepted as non-CKD. For patients who developed AKI during hospitalization, we used the KDIGO criteria for diagnosis and staging of AKI. AKI staging was performed according to the peak creatinine value. CKD was considered when the estimated glomerular filtration rate (eGFR) was < 60 mL/dk/1.73 m^2^ by the CKD-EPI formula [32].

The underlying cause of AKI was determined from clinical and other laboratory findings and/or the follow-up of the renal function. If renal recovery has occurred within 3 days of fluid replacement, prerenal AKI was considered. In addition, AKI due to cardiac decompensation without any obvious cause(s) was accepted as prerenal AKI if it resolved over a short period. Renal AKI was considered in patients with sepsis or shock or severe and prolonged prerenal AKI, with a diagnosis of acute tubular necrosis, and exposure to nephrotoxic drugs or radiocontrast medium was regarded as nephrotoxic AKI if there was no other identified cause.

### Outcomes

We defined length of stay at the hospital as the period from the first day of hospitalization to the day of discharge or death. The primary outcomes were patient and renal outcomes. Patient outcome was in-hospital death. Renal outcomes were categorized as follows: (1) Complete recovery: serum creatinine level decreased to baseline or to within normal limits; (2) partial recovery: serum creatinine level decreased but higher than 30% above baseline or reference value; (3) no improvement and/or dialysis dependence. Renal outcomes were determined only for discharged patients at the end of the hospital stay.

### Statistical analysis

The analyses were performed using the IBM SPSS Statistics for Windows, Version 25.0 (IBM Corp., Armonk, NY, USA). Descriptive statistics were expressed as numbers and percentages for categorical variables and as median and interquartile range (IQR) for numerical variables. The conformity of variables to normal distribution was assessed using visual (histogram and probability graphs) and analytical methods (Kolmogorov-Smirnov/Shapiro-Wilk tests). For multiple group comparisons of categorical variables, chi-square test or Fisher’s exact test were used for categorical variables as appropriate. In comparison of two independent groups, Mann-Whitney U test was used for non-normally distributed numerical variables. The Kruskal–Wallis test was used for non-normally distributed numerical variables in multiple group comparisons. In multivariate Cox regression analysis, we assessed interactions of AKI development during hospitalization with demographic and clinic parameters that suggested potential effect in univariate analysis. Independent risk factors of in-hospital mortality were assessed using multivariate Cox regression analysis. We checked the correlations and interactions between explanatory variables which might have potential effect on in-hospital mortality in the univariate analysis or when clinically relevant. Final multivariate models were derived using stepwise backward LR method from the initial model created with the candidate variables in Cox regression analysis. Statistical significance level was set at p<0.05.

## Results

### Clinical characteristics

The demographic, laboratory, and clinical data of the patients, according to AKI stage, are listed in Table 1. The patients’ median age was 69 (59–77) years, and 60.9% were male. The most frequent baseline comorbidities were hypertension (70.5%), diabetes mellitus (43.8%), and chronic kidney disease (37.6%). About 53% of the patients were on angiotensin-converting enzyme inhibitor (ACEI) or angiotensin receptor blocker (ARB) therapy. The rates of mild-to-moderate, severe, and critical disease at hospital admission were 33.9%, 44.3%, and 19.6%, respectively. A total of 291 patients (50.3%) were admitted to the ICU, among whom 80.6% and 6.8% were intubated and treated with ECMO, respectively. Fifty-three of 260 (20.4%) patients admitted to ICU required KRT for any indication.

Among the 578 patients with AKI, 312 (54.0%), 143 (24.7%) and 123 (21.3%) had stage 1, 2, and 3 AKI, respectively (Table 1). The prevalence of critical disease was higher in patients with stage 3 AKI at admission. The baseline serum AST, LDH, ferritin, and procalcitonin levels were higher and the baseline albumin, hemoglobin, blood lymphocyte, and platelet counts were lower in patients with AKI stage 3. AKI stage 3 was more common in patients admitted to ICU and the need for mechanical ventilation was also higher than other stages. ECMO and slow continuous dialysis treatment were also higher in these patients. Unfavorable laboratory clinical findings including lymphopenia, anemia, increase in LDH (greater than twofold the upper limit of normal [ULN]) and aspartate aminotransferase (AST) (greater than twofold the ULN), MAS, shock/severe hypotension, and secondary bacterial infection were more common in patients with AKI stage 3 at any time during hospitalization. Intrarenal etiologies of AKI were more frequent in patients with stage 3 AKI compared to those with AKI stage 1 or 2.

#### The timing of initial development of AKI

The demographic and clinical characteristics of the patients according to the timing of AKI diagnosis are shown on S2 Table. Preexisting CKD or RAAS blockage usage was more common in patients who were diagnosed with AKI at hospital admission, whereas those with baseline hypertension developed AKI more frequently during their hospital stay. The incidence of lymphopenia, macrophage activation syndrome, shock/severe hypotension, and intensive care unit admission was higher in patients who developed AKI during hospitalization. In-hospital mortality rate were increased in those who developed AKI during hospital stay. In Cox-regression analysis, intensive care admission and lymphopenia were independently associated with AKI developed during hospitalization (S3 Table).

#### AKI patients with baseline chronic kidney disease

The demographic, clinical, and laboratory data according to baseline CKD are listed in Table 2 and S4 Table. Sixty-three patients who had no prior serum creatinine measurement and showed full renal recovery at the time of discharge were considered as “non-CKD”. Among the 510 patients, 192 (37.6%) had CKD at baseline.

**Table 2:** Characteristics and outcomes of COVID-19 RT-PCR positive patients on the basis of baseline chronic kidney disease.

Variable		Baseline Chronic Kidney Disease		
	Total (n = 510)	No (n = 318)	Yes (n = 192)	p
**Age (years)**	69 (59–77.2)	64 (54–74)	72.5 (65–79)	<0.001
**Male, n/N (%)**	305/510 (59.8)	208/318 (65.4)	97/192 (50.2)	0.001
**Comorbid conditions, n/N (%)**				
Diabetes mellitus	226/506 (44.7)	119/315 (37.8)	107/191 (56.0)	<0.001
Hypertension	362/502 (72.1)	190/310 (61.3)	172/192 (89.6)	<0.001
Obesity	24/429 (5.6)	10/248 (4.0)	14/181 (7.7)	0.135
Chronic obstructive pulmonary disease	83/489 (17.0)	38/303 (12.5)	45/186 (24.2)	0.001
Coronary heart disease	159/480 (33.1)	73/299 (24.4)	86/181 (47.5)	<0.001
Heart failure	79/470 (16.8)	29/296 (9.8)	50/174 (28.7)	<0.001
Cerebrovascular disease	36/488 (7.4)	19/303 (6.3)	17/185 (9.2)	0.284
Cancer	61/495 (12.3)	45/310 (14.5)	16/185 (8.6)	0.066
Chronic liver disease	10/495 (2.0)	8/308 (2.6)	2/187 (1.1)	0.332
Autoimmune/autoinflammatory disease	24/495 (4.8)	20/309 (6.5)	4/186 (2.2)	0.031
**Medications, n/N (%)**				
ACE-I or ARB	246/460 (53.5)	136/276 (49.3)	110/184 (59.8)	0.029
Ca antagonists	159/450 (35.3)	76/266 (28.6)	83/184 (45.1)	<0.001
Beta blockers	205/449 (45.7)	96/265 (36.2)	109/184 (59.2)	<0.001
Other antihypertensives	97/433 (22.4)	46/261(17.6)	51/172 (29.7)	0.005
Insulin	117/446 (26.2)	42/262 (16.0)	75/184 (40.8)	<0.001
Oral antidiabetics	120/447 (26.8)	73/264 (27.7)	47/183 (25.7)	0.666
Statins	82/439 (18.7)	31/259 (12.0)	51/180 (28.3)	<0.001
Antiaggregant or anticoagulant drugs	232/455 (51.0)	99/267 (37.1)	133/188 (70.7)	<0.001
**Smoking status, n/N (%)** 0.002				
Never	207/386 (53.6)	120/223 (53.8)	87/163 (53.4)	
Current	27/386 (7.0)	24/223 (10.8)	3/163 (1.8)	
Former	152/386 (39.4)	79/223 (35.4)	73/163 (44.8)	
**Symptoms related to COVID-19, n/N (%)**				
Fever	345/500 (69.0)	201/310 (64.8)	144/199 (75.8)	0.013
Fatigue	325/500 (65.0)	186/310 (60.0)	139/190 (73.2)	0.003
Dyspnea	331/505 (65.5)	194/314 (61.8)	137/191 (71.7)	0.026
Cough	366/500 (73.2)	220/309 (71.2)	146/191 (76.4)	0.213
Anorexia	129/481 (26.8)	66/299 (22.1)	63/182 (34.6)	0.003
Myalgia	179/482 (37.1)	95/298 (32.0)	84/194 (44.8)	0.003
Headache	72/484 (14. 9)	39/300 (13.0)	33/184 (17.9)	0.149
Sore throat	67/485 (13.8)	33/301 (11.0)	34/184 (18.5)	0.022
Diarrhea	29/489 (5.9)	23/303 (7.6)	6/186 (3.2)	0.050
Others	29/449 (6.5)	25/286 (8.7)	4/163 (2.5)	0.009
Asymptomatic	4/457 (0.9)	3/286 (1.0)	1/171 (0.6)	-
**Oxygen saturation in room air at diagnosis, n/N (%)** 0.026				
Normal	120/489 (24.5)	80/299 (26.8)	40/190 (21.1)	
90–95%	195/489 (39.9)	105/299 (35.1)	90/190 (47.4)	
<90%	174/489 (35.6)	114/299 (38.1)	60/190 (31.6)	
**Severity of COVID-19 infection, n/N (%)** 0.649				
Asymptomatic	13/510 (2.5)	9/318 (2.8)	4/192 (2.1)	
Mild to moderate	180/510 (36.1)	120/318 (37.7)	64/192 (33.3)	
Severe	220/510 (43.1)	131/318 (41.2)	89/192 (46.4)	
Critical	93/510 (18.2)	58/318 (18.2)	35/192 (18.2)	
**Data of renal function within the last year.**				
Serum creatinine (μmol/L)	85.8 (70.7–109.6)	72.5 (62.8–81.3)	120.2 (97.3–161.8)	<0.001
eGFR (mL/min/1.73 m^2^)	65.4 (40.6–96.6)	91.3 (72.2–112)	36.4 (23.9–52.2)	<0.001
**Laboratory parameters at hospital admission**				
Urea (mmol/L)	9.3 (6.0–14.0)	7.5 (5.3–11.0)	13.0 (8.8–18.8)	<0.001
Creatinine (μmol/L)	117.6 (88.4–164.5)	100.4 (79.6–132.6)	159.6 (123.8–236.5)	<0.001
Na (mmol/L)	137 (134–140)	137 (134–140)	137 (134–140)	0.708
K (mmol/L)	4.4 (3.9–4.8)	4.2 (3.8–4.6)	4.6 (4.1–5)	<0.001
AST (U/L)	32 (20–49.4)	34 (21.8–52.7)	25 (18–40)	<0.001
ALT (U/L)	23 (14–35)	25 (16–38)	18 (12–30)	<0.001
LDH (U/L)	320 (239–443)	334 (239–574.2)	298 (237.2–376.5)	<0.001
Albumin (g/L)	34.7 (30–38.9)	35 (30–39.4)	35 (30.8–38)	0.411
Ferritin (μg/L)	260 (160.5–750)	371 (162.2–778.2)	309 (154.5–636.4)	0.147
Fibrinogen (g/L)	4.7 (3.5–6.2)	4.9 (3.9–6.3)	4.3 (2.9–5.9)	<0.001
D-dimer (mg/L)	15.1 (8.1–27.9)	14.1 (7.7–31.0)	14.8 (8.5–24.7)	0.929
Procalcitonin (ng/L)	350 (130–1147.5)	355 (110–1140)	260 (130–1065)	0.861
Hemoglobin (g/dl)	12.1 (10.6–13.8)	12.7 (11–14.2)	11.4 (10.1–13)	<0.001
Leucocyte count (/mm3)	7835 (5500–11262)	7540 (5400–10737)	8490 (6240–11800)	0.014
Neutrophil count (/mm3)	5750 (3700–9110)	5400 (3500–9070)	6500 (4190–9230)	0.023
Lymphocyte count (/mm3)	1100 (700–1520)	1120 (740–1600)	1100 (720–1500)	0.526
Thrombocyte count (x1000/mm3)	202.5 (151–274.5)	200.5 (152–276.5)	217.5 (154.5–283.2)	0.327
CRP levels^†^, n/N (%)				0.356
Normal	28/509 (5.5)	19/317 (6.0)	9/192 (4.7)	
1/5-fold x ULN	83/509 (16.3)	57/317 (18.0)	26/192 (13.5)	
5/10-fold x ULN	94/509 (18.5)	62/317 (19.6)	32/192 (16.7)	
10/20-fold x ULN	136/509 (26.7)	77/317 (24.3)	59/192 (30.7)	
>20-fold x ULN	168/509 (33.0)	102/317 (32.2)	66/192 (34.4)	
**Unfavorable prognostic signs at any time during hospital stay, n/N (%)**				
Lymphopenia	387/510 (75.9)	240/318 (75.5)	147/192 (76.6)	0.831
Anemia (Hb <10 g/dL)	248/510 (48.6)	150/318 (47.2)	98/192 (51.0)	0.412
Thrombocytopenia	161/506 (31.8)	102/316 (32.3)	59/190 (31.1)	0.844
LDH (>2-fold x ULN) ^‡^	257/493 (52.1)	171/307 (55.7)	86/186 (46.2)	0.051
AST (>2-fold x ULN) ^‡‡^	212/507 (41.8)	154/316 (48.7)	58/191 (30.4)	<0.001
Macrophage activation syndrome	104/472 (22.0)	72/287 (25.1)	32/185 (17.3)	0.053
Shock/severe hypotension	167/492 (33.9)	110/302 (36.4)	57/190 (30.0)	0.171
Secondary bacterial infection	208/476 (43.7)	128/292 (43.9)	80/184 (43.5)	1.000
CRP levels^†^, n/N (%)				0.940
Normal	17/510 (3.3)	12/318 (3.8)	5/192 (2.6)	
1/5-fold x ULN	38/510 (7.5)	23/318 (7.2)	15/192 (7.8)	
5/10-fold x ULN	56/510 (11.0)	34/318 (10.7)	22/192 (11.5)	
10/20-fold x ULN	114/510 (22.4)	73/318 (23.0)	41/192 (21.4)	
>20-fold x ULN	285/510 (55.9)	176/318 (55.3)	109/192 (56.8)	
**Intensive care unit admission, n/N (%)**	239/510 (46.9)	150/318 (47.2)	89/192 (46.4)	0.927
**Managements in the intensive care unit, n/N (%)**				
Intubation	182/237 (76.8)	116/148 (78.4)	66/89 (74.2)	0.525
ECMO	16/223 (7.2)	8/137 (5.8)	8/86 (9.3)	0.425
Slow continuous dialysis	39/221 (17.6)	22/135 (16.3)	17/86 (19.8)	0.588
**Duration of stay in intensive care unit (days)**	9 (5.8–17)	10 (6–19)	8 (5–16)	0.115
**AKI timing, n/N (%)**				
AKI at hospital admission	225/510 (44.1)	125/318 (39.3)	100/192 (52.1)	0.006
AKI during hospital stay	285/510 (55.9)	193/318 (60.7)	92/192 (47.9)	0.006
**Suspected causes of AKI, n/N (%)** 0.222				
Prerenal	229/510 (44.9)	151/318 (47.5)	78/192 (40.6)	
Renal	269/510 (52.7)	158/318 (49.7)	111/192 (57.8)	
Postrenal	5/510 (1.0)	3/318 (0.9)	2/192 (1.0)	
Others	7/510 (1.4)	6/318 (1.9)	1/192 (0.5)	
**Suspected specific causes of AKI, n/N (%)** 0.136				
Dehydration	134/510 (26.3)	82/318 (25.8)	52/192 (27.1)	
GIS loss	9/510 (1,8)	6/318 (1.9)	3/192 (1,6)	
Heart failure	15/510 (2.9)	9/318 (2.8)	6/192 (3,1)	
Other prerenal causes	71/510 (13,9)	54/318 (17.0)	17/192 (8.9)	
Sepsis	202/510 (39.6)	112/318 (35.2)	90/192 (46.9)	
Thrombotic microangiopathy	4/510 (0.8)	4/318 (1,3)	0/192 (0)	
Extended prerenal causes	32/510 (6,3)	21/318 (6.6)	11/192 (5.7)	
Rhabdomyolysis	2/510 (0,4)	1/318 (0,3)	1/192 (0,5)	
Nephrotoxic drugs	29/510 (5,7)	20/318 (6,3)	9/192 (4,7)	
Postrenal (urological) causes	5/510 (1.0)	3/318 (0,9)	2/192 (1.0)	
Others	7/510 (1,4)	6/318 (1,9)	1/192 (0.5)	
**AKI Stage, n/N (%)** 0.287				
Stage 1	289/510 (56.7)	186/318 (58.5)	103/192 (53.6)	
Stage 2	127/510 (24.9)	80/318 (25.2)	47192 (24.5)	
Stage 3	94/510 (18.4)	52/318 (16.4)	42/192 (21.9)	
**Time between hospitalization and AKI diagnosis (days)**	5 (3–8)	5 (2.3–8)	5 (3–7)	0.654
**Dialysis requirement in the ward, n/N (%)**	77/490 (15.7)	34/289 (11.8)	43/201 (21.4)	0.005
**KRT indications, n/N (%)** 0.185				
Increase in serum BUN/creatinine levels	32/94 (34.0)	16/46 (34.8)	16/48 (33.3)	
Hyperkalemia	8/94 (8.5)	6/46 (13.0)	2/48 (4.2)	
Metabolic acidosis	24/94 (25.5)	14/46 (30.4)	10/48 (20.8)	
Hypervolemia	18/94 (19.1)	5/46 (10.9)	13/48 (27.1)	
Severe uremic symptoms	3/94 (3.2)	2/46 (4.3)	1/48 (2.1)	
Others	9/94 (9.6)	3/46 (6.5)	6/48 (12.5)	
**Renal Outcome, n/N (%)**				<0.001
Complete recovery	285/333 (85.6)	192/209 (91.9)	93/124 (75)	
Partially recovery	46/333 (13.8)	17/209 (8.1)	29/124 (23.4)	
Dialysis dependence	2/333 (0.6)	0/209 (0)	2/124 (1.6)	
**Duration of AKI in discharged patients (days)**	6 (3–9)	5 (3–8)	7 (4–10)	<0.001
**Patient Outcome, n/N (%)**				
Discharged	336/510 (65.9)	210/318 (66.0)	126/192 (65.6)	
Dead	174/510 (34.1)	108/318 (34.0)	66/192 (34.4)	
**Total hospital stays (days)**	12 (8–19)	12 (8–20)	12 (8–18)	0.446

ACE-I, angiotensin-converting enzyme inhibitors; ARB, angiotensin receptor blockers; COVID-19 coronavirus disease 2019; eGFR, estimated glomerular filtration rate; AST, aspartate aminotransferase; ALT, alanine aminotransferase; LDH, lactate dehydrogenase; x ULN, increase above upper normal limit; CRP, C-reactive protein; ECMO, extracorporeal membrane oxygenation; KRT, kidney replacement therapy.

Data were expressed as median [Q1-Q3] or as number (percent).

^†^The upper limit of the normal range of CRP was 5 mg/L (47.6 nmol/L).

^‡^The upper limit of the normal range of LDH was 248 U/L.

^‡‡^The upper limit of the normal range of AST was 37 U/L.

In patients with an eGFR level in the last year (n = 447), the prevalence of CKD stage 3, 4, and 5 was 25.3%, 12.3%, and 5.1%, respectively. Patients who had CKD were older and had more comorbidities, including diabetes mellitus, hypertension, chronic obstructive pulmonary disease, and heart disease.

The incidence of AKI at admission was higher in patients with CKD than those without CKD (52.1% *vs*. 39.3%, respectively, p = 0.006). The requirement for hemodialysis in CKD patients followed on the ward was more frequent, whereas intensive care admission and slow continuous dialysis rates were not different compared to non-CKD patients (Table 2).

### Survival

The demographic and clinical characteristics of discharged and deceased patients are listed in Table 3 and S5 Table. There were 225 (38.9%) deaths in the overall cohort. During hospitalization, 34.4% of patients with CKD and 34.0% of patients without CKD died. The in-hospital mortality rate was 18.9% (n = 59), 46.2% (n = 66), and 81.3% (n = 100) among those with AKI stages 1, 2, and 3, respectively.

**Table 3:** Characteristics of COVID-19 RT-PCR positive patients based on patient survival.

Variable	Total (n = 578)	Discharged (n = 353)	Dead (n = 225)	p
**Age (years)**	69 (59–77)	67 (57–77)	72 (63–78)	0.001
**Male, n/N (%)**	352/578 (60.9)	203/353 (57.5)	149/225 (66.2)	0.036
**Comorbid conditions, n/N (%)**				
Diabetes mellitus	249/569 (43.8)	143/349 (41)	106/220 (48.2)	0.091
Hypertension	399/566 (70.5)	256/346 (74)	143/220 (65)	0.022
Chronic kidney disease	192/510 (37.6)	126/336 (37.5)	66/174 (37.9)	0.924
Obesity	27/462 (5.8)	18/292 (6.2)	9/170 (5.3)	0.701
Chronic obstructive lung disease	89/549 (16.2)	53/343 (15.5)	36/206 (17.5)	0.533
Coronary heart disease	172/541 (31.8)	101/338 (29.9)	71/203 (35)	0.218
Heart failure	85/528 (16.1)	52/331 (15.7)	33/197 (16.8)	0.753
Cerebrovascular disease	39/546 (7.1)	15/341 (4.4)	24/205 (11.7)	0.001
Cancer	69/554 (12.5)	26/342 (7.6)	43/212 (20.3)	<0.001
Chronic liver disease	10/556 (1.8)	5/343 (1.5)	5/213 (2.3)	0.518
Autoimmune/autoinflammatory disease	25/556 (4.5)	15/343 (4.4)	10/213 (4.7)	0.859
**Medications, n/N (%)**				
ACE-I or ARB	266/505 (52.7)	193/321 (60.1)	73/184 (39.7)	<0.001
Ca antagonists	171/491 (34.8)	113/309 (36.6)	58/182 (31.9)	0.291
Beta blockers	216/491 (44)	141/309 (45.6)	75/182 (41.2)	0.340
Other antihypertensives	113/476 (23.7)	70/304 (23)	43/172 (25)	0.627
Insulin	122/484 (25.2)	73/304 (24)	49/180 (27.2)	0.432
Oral antidiabetics	127/486 (26.1)	82/304 (27)	45/182 (24.7)	0.585
Statins	88/477 (18.4)	48/302 (15.9)	40/175 (22.9)	0.059
Antiaggregant or anticoagulant drugs	251/501 (50.1)	152/312 (48.7)	99/189 (52.4)	0.427
**Smoking status, n/N (%)** 0.488				
Never	221/411 (53.8)	148/265 (55.8)	73/146 (50)	
Current	34/411 (8.3)	20/265 (7.5)	14/146 (9.6)	
Former	156/411 (38)	97/265 (36.6)	59/146 (40.4)	
**Symptoms related to COVID-19, n/N (%)**				
Fever	380/565 (67.3)	235/351 (67)	145/214 (67.8)	0.843
Fatigue	356/563 (63.2)	228/347 (65.7)	128/216 (59.3)	0.123
Dyspnea	375/569 (65.9)	206/351 (58.7)	169/218 (77.5)	<0.001
Cough	408/564 (72.3)	255/349 (73.1)	153/215 (71.2)	0.624
Anorexia	132/540 (24.4)	87/339 (25.7)	45/201 (22.4)	0.392
Myalgia	192/542 (35.4)	129/343 (37.6)	63/199 (31.7)	0.163
Headache	82/545 (15)	50/343 (14.6)	32/202 (15.8)	0.690
Sore throat	72/546 (13.2)	52/344 (15.1)	20/202 (9.9)	0.082
Diarrhea	32/551 (5.8)	25/347 (7.2)	7/204 (3.4)	0.067
Others	40/508 (7.9)	23/323 (7.1)	17/185 (9.2)	0.405
Asymptomatic	4/519 (0.8)	4/324 (1.2)	0/195 (0)	
**Radiologic examination, n/N (%)**				
Patients with a chest CT scan	563/572 (98.4)	349/351 (99.4)	214/221 (96.8)	0.031
Patients with specific chest CT findings	529/564 (93.8)	327/350 (93.4)	202/214 (94.4)	0.645
Patients with specific bilaterally chest CT findings	472/549 (86)	282/341 (82.7)	190/208 (91.3)	0.005
**Specific chest CT findings, n/N (%)**				
Ground glass opacity	503/535 (94)	307/330 (93)	196/205 (95.6)	0.221
Reticular opacity	155/428 (36.2)	98/270 (36.3)	57/158 (36.1)	0.963
Bronchial wall thickening	79/428 (18.5)	49/270 (18.1)	30/158 (19)	0.829
Pleural effusion	100/427 (23.4)	52/269 (19.3)	48/158 (30.4)	0.009
Thoracic lymphadenopathy	53/424 (12.5)	35/268 (13.1)	18/156 (11.5)	0.648
**Oxygen saturation in room air at diagnosis, n/N (%)**	<0.001			
Normal	129/551 (23.4)	111/335 (33.1)	18/216 (8.3)	
90–95%	210/551 (38.1)	155/335 (46.3)	55/216 (25.5)	
<90%	212/551 (38.5)	69/335 (20.6)	143/216 (66.2)	
**Severity of COVID-19 infection** <0.001				
Asymptomatic	13/578 (2.2)	12/353 (3.4)	1/225 (0.4)	
Mild to moderate	196/578 (33.9)	169/353 (47.9)	27/225 (12)	
Severe	256/578 (44.3)	145/353 (41.1)	111/225 (49.3)	
Critical	113/578 (19.6)	27/353 (7.6)	86/225 (38.2)	
**Data for renal function within the last year**				
Serum creatinine (μmol/L)	88.4 (70.7–106.1)	88.4 (70.7–123.8)	79.6 (70.7–106.1)	0.010
eGFR (mL/min/1.73 m^2^)	65.3 (40.6–96.6)	63.1 (36.9–90.3)	74.7 (48.7–102.1)	0.005
**CKD stages in patients with known eGFR** 0.040 **within the last year (n = 317), n/N (%)**				
eGFR>90 ml/min/1.73m2	130/447 (29.1)	67/273 (24.5)	63/174 (36.2)	
eGFR 60–90 ml/min/.73m^2^	126/447 (28.2)	/273 (29.3)	46/174 (26.4)	
CKD stage 3	113/447 (25.3)	69/273 (25.3)	44/174 (25.3)	
CKD stage 4	55/447 (12.3)	40/273 (14.7)	15/174 (8.6)	
CKD stage 5	23/447 (5.1)	17/273 (6.2)	6/174 (3.4)	
CKD stage 4 and 5 (eGFR<30 ml/dk/1.73 m^2^)	78/578 (13.5)	57/353 (16.1)	21/225 (38.9)	0.024
**Laboratory parameters at hospital admission**				
Urea (mmol/L)	9.3 (6.0–14.0)	9.2 (6.0–13.6)	9.3 (6.0–14.8)	0.783
Creatinine (μmol/L)	117.6 (88.4–164.4)	123.8 (95.5–160.9)	106.1 (79.6–168.0)	0.005
Na (mmol/L)	137 (134–140)	137 (134–140)	136 (133–140)	0.357
K (mmol/L)	4.4 (3.9–4.8)	4.4 (4–4.8)	4.245 (3.8–4.7)	0.009
AST (U/L)	32 (20–49.4)	26.5 (19–40)	41 (27–66)	<0.001
ALT (U/L)	23 (14–35)	21 (14–32.5)	24.55 (14–43)	0.014
LDH (U/L)	320 (239–443)	285 (224–383)	383 (297–570)	<0.001
Albumin (g/L)	34.7 (30–38.9)	37.0 (32.6–39.8)	31.0 (28.0–35.0)	<0.001
Ferritin (μg/L)	360 (162–748)	267 (137–632)	511.35 (286–968)	<0.001
Fibrinogen (g/L)	4.8 (3.5–6.2)	4.5 (3.3–6.0)	4.9 (3.9–6.3)	0.029
D-dimer (mg/L)	14.6 (8.1–27.9)	13.5 (7.9–24.0)	15.1 (9.3–33.1)	0.056
Procalcitonin (ng/L)	350 (130–114.5)	230 (100–780)	830 (220–1780)	<0.001
Hemoglobin (g/dl)	12.1 (10.6–13.8)	12.45 (11–14)	11.8 (10–13.3)	<0.001
Leucocyte count(/mm3)	7835 (5500–11250)	7420 (5370–10200)	8700 (5900–12290)	0.003
Neutrophil count (/mm3)	5750 (3700–9110)	5300 (3600–7810)	7100 (4200–10310)	<0.001
Lymphocyte count (/mm3)	1100 (700–1520)	1200 (900–1600)	885 (580–1400)	<0.001
Thrombocyte count (x1000/mm3)	202.5 (151–274)	209.5 (160,6–280)	190 (138–266)	0,016
CRP levels^†^, n/N (%) <0.001				
Normal	28/576 (4,9)	26/352 (7,4)	2/224 (0,9)	
1/5-fold x ULN	91/576 (15,8)	76/352 (21,6)	15/224 (6,7)	
5/10-fold x ULN	103/576 (17,9)	72/352 (20,5)	31/224 (13,8)	
10/20-fold x ULN	150/576 (26)	89/352 (25,3)	61/224 (27,2)	
>20-fold x ULN	204/576 (35,4)	89/352 (25,3)	115/224 (51,3)	
**Unfavorable prognostic signs at any time during hospital stay, n/N (%)**				
Lymphopenia	88/577 (15.3)	47/353 (13.3)	41/224 (18.3)	0.104
Anemia (Hb <10 g/dL)	443/578 (76.6)	249/353 (70.5)	194/225 (86.2)	<0.001
Thrombocytopenia	293/578 (50.7)	135/353 (38.2)	158/225 (70.2)	<0.001
LDH (>2-fold x ULN) ^‡^	189/574 (32.9)	82/353 (23.2)	107/221 (48.4)	<0.001
AST (>2-fold x ULN) ^‡‡^	303/559 (54.2)	142/347 (40.9)	161/212 (75.9)	<0.001
Macrophage activation syndrome	254/575 (44.2)	102/353 (28.9)	152/222 (68.5)	<0.001
Shock/severe hypotension	124/519 (23.9)	32/340 (9.4)	92/179 (51.4)	<0.001
Secondary bacterial infection	213/557 (38.2)	21/342 (6.1)	192/215 (89.3)	<0.001
CRP levels^†^, n/N (%)				<0.01
Normal	17/578 (2.9)	17/353 (4.8)	0/225 (0)	
1/5-fold x ULN	40/578 (6.9)	38/353 (10.8)	2/225 (0.9)	
5/10-fold x ULN	59/578 (10.2)	51/353 (14.4)	8/225 (3.6)	
10/20-fold x ULN	124/578 (21.5)	90/353 (25.5)	34/225 (15.1)	
>20-fold x ULN	338/578 (58.5)	157/353 (44.5)	181/225 (80.4)	
**Intensive care unit admission, n/N (%)**	291/578 (50.3)	72/353 (20.4)	219/225 (97.3)	<0.001
**Managements in the intensive care unit, n/N (%)**				
Intubation	232/288 (80.6)	25/72 (34.7)	207/216 (95.8)	<0.001
ECMO	18/543 (3.3)	0/70 (0)	18/195 (9.2)	0.003
Slow continuous dialysis	53/260 (20.4)	9/71 (12.7)	44/159 (23.3)	0.039
**Duration of stay in intensive care unit (days)**	9 (6–17)	8.5 (6–16)	9 (5–17)	0.965
**AKI timing, n/N (%)**				
AKI at hospital admission	251/578 (43.4)	171/251 (68.1)	80/251 (31.9)	0.003
AKI during hospital stay	327/578 (56.6)	182/327 (55.7)	145/327 (44.3)	0.003
**AKI Stage, n/N (%)** <0.001				
Stage 1	312/578 (54)	253/353 (71.7)	59/225 (26.2)	
Stage 2	143/578 (24.7)	77/353 (21.8)	66/225 (29.3)	
Stage 3	123/578 (21.3)	23/353 (6.5)	100/225 (44.4)	
**Suspected causes of AKI, n/N (%)**				<0.001
Prerenal	251/578 (43.4)	220/353 (62.3)	31/225 (13.8)	
Renal	311/578 (53.8)	124/353 (35.1)	187/225 (83.1)	
Postrenal	6/578 (1)	5/353 (1.4)	1/225 (0.4)	
Others	10/578 (1.7)	4/353 (1.1)	6/225 (2.7)	
**Time between hospitalization and AKI diagnosis (days)**	5 (3–8)	4 (2–7)	5 (3–9)	0.304
**Dialysis requirement in the ward, n/N (%)**	91/554 (16.4)	10/343 (2.9)	81/211 (38.4)	<0.001
**Total hospital stays (days)**	12 (8–19)	12 (8–17)	13 (9–21)	0.103

ACE-I, angiotensin-converting enzyme inhibitors; ARB, angiotensin receptor blockers; COVID-19, coronavirus disease 2019; eGFR, estimated glomerular filtration rate; AST, aspartate aminotransferase; ALT, alanine aminotransferase; LDH, lactate dehydrogenase; x ULN, increase above upper normal limit; CRP, C-reactive protein; ECMO, extracorporeal membrane oxygenation.

Data were expressed as median [Q1-Q3] or as number (percent).

^†^The upper limit of the normal range of CRP was 5 mg/L (47.6 nmol/L).

The upper limit of the normal range of LDH was 248 U/L.

^‡‡^The upper limit of the normal range of AST was 37 U/L.

In the multivariate Cox regression analysis; age, male gender, diabetes, and cerebrovascular disease, LDH (greater than twofold the ULN), and AKI stages were independently associated with in-hospital mortality (Table 4). Adjusted in-hospital mortality was increased in patients with AKI stage 2 (HR 1.98, 95%CI 1.25–3.14, p = 0.003) and stage 3 (HR 2.25, 95%CI 1.44–3.51, p = 0.0001) compared to those with AKI stage 1.

**Table 4:** Covariates associated with death by Cox survival analysis.

Variables	Univariate Analysis			Multivariate Analysis		
	HR	95% CI	p	HR	95% CI	P
Age (years)	1.01	0.99–1.03	0.068	1.01	1.00–1.03	0.035
Male gender	1.46	1.01–2.11	0.042	1.47	1.04–2.09	0.029
Diabetes mellitus	1.55	1.03–2.32	0.034	1.51	1.06–2.17	0.022
Hypertension	0.94	0.62–1.43	0.797			
Cerebrovascular disease	1.90	1.11–3.26	0.019	1.82	1.08–3.07	0.023
Malignancy	1.12	0.70–1.78	0.631			
Oxygen saturation in room air at hospital admission (<90%)	1.25	0.68–2.29	0.463			
eGFR in previous year (ml/min/1.73m^2^)	0.99	0.99–1.00	0.402			
Serum albumin (g/L)	0.74	0.54–1.03	0.082	0.74	0.54–1.01	0.064
CRP (>20-fold x ULN)	0.88	0.61–1.26	0.494			
LDH (>2-fold x ULN)	1.45	0.95–2.20	0.079	1.55	1.05–2.30	0.027
Lymphocyte count (mm^3^)	1.00	1.00–1.00	0.708			
Thrombocyte count (mm^3^)	0.99	0.99–1.00	0.047	0.99	0.99–1.00	0.052
AKI stage 1 (ref)						
AKI stage 2	1.85	1.15–2.98	0.011	1.98	1.25–3.14	0.003
AKI stage 3	2.19	1.40–3.43	<0.001	2.25	1.44–3.51	<0.001

CRP, C-reactive protein; LDH, lactate dehydrogenase.

**Fig 2:**
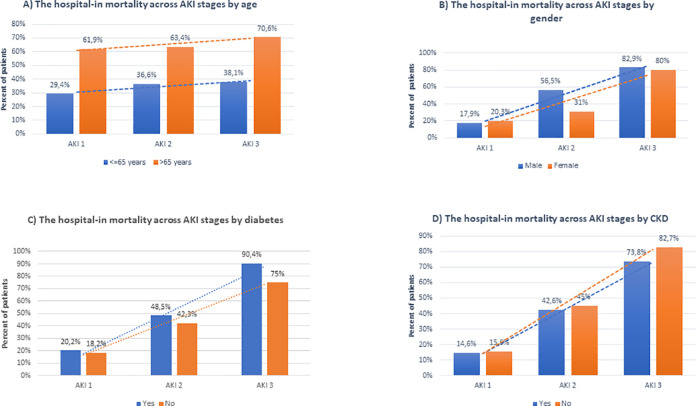
The in-hospital mortality rate across acute kidney injury (AKI) stages by age (A), gender (B), diabetes mellitus (C), and chronic kidney disease (CKD) (D).

The in-hospital mortality rates across AKI stages by age, gender, diabetes mellitus, and CKD were shown in Fig 2.

#### Renal outcomes

Patients who died during hospitalization were excluded from the analysis of renal outcomes (S6 Table). Renal improvement was complete in 81.7% (285 of 349 patients) and partial in 17.2% (60 of 349 patients) of the patients. In 1.1% of the patients (4 of 349 patients), there was no improvement in renal function (n = 1) or the patient remained dialysis-dependent (n = 3) at discharge.

The complete recovery rate was 84.2%, 76.6%, and 56.5% in AKI stages 1, 2, and 3, respectively (S3 Table). The complete recovery rate was 75.0% and 91.9% in patients with CKD and non-CKD, respectively (p = 0.042). Although partial recovery was more frequent in patients with CKD (23.4% *vs*. 8.1%, p = 0.010), few patients were dialysis-dependent at discharge (0 [0%] and two [1.6%]) patients in the non-CKD and CKD groups, respectively) (Table 2). In a patient who was dialysis-dependent at discharge, baseline CKD status was unknown.

## Discussion

In this multicenter retrospective study, we documented the characteristics and renal and patient outcomes of 578 hospitalized patients with COVID-19 and AKI. The in-hospital mortality rate was remarkably high in these patients and increased with AKI stage. Older age, male gender, baseline diabetes mellitus and cerebrovascular disease, increased LDH, and severity of AKI were independently associated with mortality. The renal outcomes were also worse in patients with prior CKD and those with AKI stage 3.

The incidence of AKI among patients with COVID-19 and its association with outcomes were assessed in a systematic review and meta-analysis that included 20 cohorts covering 13,137 patients [33]. In this analysis, the prevalence of AKI among patients with COVID-19 was 17% (range, 0.5–80%). The variance in the prevalence of AKI among the cohorts was attributed to different clinical practices and patient characteristics as well as the severity of underlying disease and/or of AKI (28). In patients with AKI, the mortality rate was estimated at 52% (range, 7–100%) [33]. Similarly, AKI was consistently associated with increased mortality, although the magnitude of the increased risk varied. The mortality rate was increased in patients with AKI compared to those without AKI (pooled OR, 15.27; 95% CI, 4.82–48.36) [33]. In this study, the mortality rate was 38.9% in patients with COVID-19 and AKI. In our cohort, 50.3% of the patients were admitted to ICU, among whom 80.6% and 6.8% were intubated and treated with ECMO, respectively.

Diabetes and hypertension were independent risk factors for AKI in patients with COVID-19. Hence, it was not surprising that these frequencies were higher in our cohort of patients with COVID-19 and AKI. However, the frequency of CKD (37.6%) was higher than that in most previous studies (5% of all patients; range, 0.6–57.1%) [33]. In our cohort, we determined the eGFR values within the last year of 447 of the 578 patients; therefore, the CKD incidence may be more precise. By contrast, the prevalence of CKD was documented in all patients (regardless of AKI) in two previous studies [5, 34], whereas our AKI cohort included only patients with acute injury-on-chronic kidney disease. In a cohort of 3235 patients with COVID-19, the CKD prevalence was significantly higher in patients with AKI compared to those without AKI (17.3% *vs*. 4.4%, respectively, p < 0.001). It is expected that patients with COVID-19 and CKD are susceptible to AKI [35].

The mortality risk is increased in patients with COVID-19 and various forms of CKD [24–27, 36]. In a study of United Kingdom study involving more than 17 million people using the OpenSAFELY health analytics platform, patients with CKD (eGFR < 60 mL/min/1.73 m^2^) had an increased risk for mortality, particularly those with advanced CKD, dialysis, or kidney transplant [27]. European Renal Association-European Dialysis and Transplant Association (ERA-EDTA) registry data and the ERA- COVID-19 Database (ERACODA) collaboration also demonstrated that dialysis and kidney transplant recipients with COVID-19 had an increased mortality risk [24, 25]. A nationwide study in Turkey revealed that the risk of ICU admission and in-hospital mortality was increased in patients with CKD stage 3–5 compared to those without kidney disease [26].

Data on the outcome of AKI in COVID-19 patients with baseline pre-dialysis CKD are sparse [35, 37]. Patients with COVID-19 plus CKD and AKI had a higher mortality rate compared to those with only AKI or CKD (74.7% *vs*. 53.8% *vs*. 67%, respectively) [35]. Peng *et al*. reported that AKI diagnosis before the onset date of any other organ dysfunction (AKI-early) was less frequent in patients with CKD [37]. In our study, the frequency of CKD stage 3, 4, and 5 non-dialysis was 25.3%, 12.3%, and 5.1%, respectively, among the patients with AKI. The mortality rates were not different in patients with CKD (even across CKD subgroups) compared to patients who did not have CKD. Previous observational studies showed that CKD may modify the impact of AKI on mortality in non-COVID era [38–41]. The Program to Improve Care in Acute Renal Disease (PICARD) study revealed the unexpected reduced in-hospital mortality rate in patients who had prior CKD among critically ill patients with AKI [39]. In a large-scale study, preexisting CKD had similar adjusted in-hospital mortality risk across AKI stages, whereas mortality or ESKD risk increased in patients with baseline CKD during follow-up after discharge [41]. In contrast, increased in-hospital death rate was reported in CKD patients with AKI stage 2 and 3, but not AKI stage 1 [42]. Underlining mechanism(s) of these associations is not clear because of observational nature of the studies. It was postulated that earlier AKI diagnosis and/or nephrology consultation may influence the medical care or attention delivered to patients with CKD which might have resulted in favorable or at least unchanged patient outcomes in this population [39, 40]. On the other hand, small increase in serum creatinine levels at hospital admission was not a risk factor for in-hospital mortality in patients with CKD stage 4 to 5 [43]. It may reflect limited clinical significance of stage 1 AKI in advanced CKD due to erratic oscillations in serum creatinine (and/or transient renal ischemia which may result in increase in serum creatinine in CKD patients who already have reduced renal reserve [42]. Furthermore, acute renal and systemic injury may be more intensive in non-CKD patients compared to patients with CKD in certain AKI stages even if the same definitions based on serum creatinine measurement for the staging of AKI are used.

Older age, male gender, and comorbidities, including diabetes and cerebrovascular disease, were independently associated with mortality in patients with COVID-19 and AKI. These are risk factors for mortality in patients with COVID-19 [44]. Notably, the severity of AKI according to the KDIGO stage, was also a risk factor for mortality in our multivariate regression analysis. Two studies have reported the distribution of AKI stages and patient outcomes by AKI stage [11, 19]. The frequencies of AKI stages were reportedly 45.7–46.5% stage 1, 22.4–22.9% stage 2 and 31.1–31.4% stage 3, and the mortality rate increased with the severity of AKI [11, 19]. In our cohort, the proportions of AKI stages were 54%, 24.7%, and 21.3% for AKI stage 1, 2, and 3, respectively.

There are few studies to document kidney outcomes in patients with AKI and COVID-19 [45, 46]. The rates of renal recovery in these studies have varied from 65% to 74.1%. In the present cohort, complete renal recovery was observed as 80.7% which was relatively higher compared to these studies. The lower frequency of AKI stage 3 (21.3%) among the AKI patients compared to other studies (approximately 42%) may explain higher rate of renal recovery in our cohort. We also observed that complete recovery rates were worse in patients with baseline CKD or AKI stage 3.

Distinct direct or indirect pathogenic mechanisms have been implicated in AKI development in patients with COVID-19 [23, 47]. Some post-mortem studies showed direct kidney invasion by the virus [48, 49], but others did not [50–52]. In our cohort, the frequencies of prerenal and renal AKI among patients with AKI were 43.4% and 53.8%, respectively. However, we did not have urinalysis results, precluding determination of the etiology of AKI. A previous study from New York suggested that 66% of patients with AKI had a prerenal AKI state according to the urine sodium level [11].

This study had several limitations. First, a matched control group of patients without AKI or patients with non-COVID-19 AKI was not included in the survival analysis. Second, we had no urinalysis data for diagnosing the cause of AKI and identifying abnormalities such as hematuria and/or proteinuria, which are frequently observed in these patients. Third, we did not have any follow-up data after discharge. Also, we could not examine the effects of medications for the treatment of COVID-19 on the results because of the lack of information on the dose and duration of administration. Finally, because this was an observational study, we cannot interpret the causality of the relationship between exposures and outcomes.

The management of COVID-19 and AKI is not significantly different from other causes of AKI. The strategies in prevention of AKI includes early fluid management in hypovolemia or fluid and vasopressor resuscitation in septic shock may reduce the risk of AKI [47]. In COVID-19 patients who already have AKI, the goals of management should be to improve patient outcomes and prevent deterioration of AKI. The management include hemodynamic optimization to correct hypovolemia or hypervolemia, glucose management, avoiding nephrotoxic drugs or radiocontrast when possible and standard caring in multiorgan failure [47]. In patients who need KRT, it is important to provide KRT resources and to begin appropriate KRT modality with adequate dose at the right time.

In conclusion, AKI is associated with remarkably high mortality among hospitalized COVID-19 patients. The severity of AKI parallel with the severity of COVID-19 and COVID-19 related in-hospital outcomes. The risk factors reportedly associated with mortality in patients with COVID-19, such as older age, male gender, and comorbidities, including diabetes mellitus, were also valid among patients with COVID-19 and AKI. Renal problems continue in a significant portion of discharged patients, though few patients depend on dialysis. As AKI stage increases, the complete recovery rate decreases. The complete recovery rate of AKI is lower in patients with CKD than in patients with non-CKD. Early management is needed to improve patient and renal outcomes in patients with COVID-19 and AKI.

## Supporting information

S1 Table Characteristics of 835 patients during hospital admission and hospital stay, by AKI severity. (DOCX)

S2 Table Characteristics of COVID-19 RT-PCR positive patients, by the timing of initial development of AKI. (DOCX)

S3 Table Predictors associated with acute kidney injury developed hospital stay by Cox regression analysis. (DOCX)

S4 Table Some characteristics of COVID-19 RT-PCR positive patients, by CKD status. (DOCX)

S5 Table Some characteristics of COVID-19 RT-PCR positive patients, by patient survival. (DOCX)

S6 Table Characteristics of COVID-19 RT-PCR positive patients according to renal outcomes. (DOCX)

S1 Dataset (SAV)
