# Supplementary material for: Characteristics and outcomes of acute kidney injury in hospitalized COVID-19 patients: A multicenter study by the Turkish society of nephrology
Source: PLoS One. 2021 Aug 10;16(8):e0256023. doi: 10.1371/journal.pone.0256023 (PMC8354466; doi:10.1371/journal.pone.0256023)
Supplement: S1 Table — (DOCX) [file pone.0256023.s001.docx]

**S1 Table. Characteristics of 835 patients with AKI during hospital admission and hospital stay, by AKI severity**

| **Variable** | **Total**  **(n=835)** | **AKI 1**  **(n=438)** | **AKI 2**  **(n=217)** | **AKI 3**  **(n=180)** | **p** |
| --- | --- | --- | --- | --- | --- |
| **Age (years)** | 70 (59-78) | 69 (58-78) | 69 (59-78) | 71 (60.5-79) | 0.628 |
| **Male, n/N (%)** | 500 (59.9) | 257 (58.7) | 128(59) | 115 (63.9) | 0.463 |
| **Comorbid conditions, n/N (%)** | | | | | |
| Diabetes mellitus | 358/819 (42.9) | 181/435 (41.6) | 96/211 (45.5) | 81/173 (46.8) | 0.420 |
| Hypertension | 580/814 (71.3) | 317/432 (73.4) | 155/210 (73.8) | 108/172 (62.8) | 0.022 |
| Chronic kidney disease | 286/727 (39.3) | 165/339 (41.4) | 65/189 (34.4) | 56/139 (40.3) | 0.263 |
| Obesity | 42/660 (6.4) | 19/358 (5.3) | 15/174 (8.6) | 8/128 (6.3) | 0.339 |
| Chronic obstructive pulmonary disease | 151/783 (19.3) | 87/421 (20.7) | 31/197 (15.7) | 33/165 (20) | 0.339 |
| Coronary heart disease | 278/771 (36.1) | 138/405 (34.1) | 80/200 (40) | 60/166 (36.1) | 0.361 |
| Heart failure | 137/746 (18.4) | 73/394 (18.5) | 41/192 (21.4) | 23/160 (14.4) | 0.240 |
| Cerebrovascular disease | 69/776 (8.9) | 33/416 (7.9) | 19/199 (9.5) | 17/161 (10.6) | 0.568 |
| Cancer | 106/791 (13.4) | 39/426 (9.2) | 35/201 (17.4) | 32/164 (19.5) | 0.001 |
| Chronic liver disease | 16/792 (2) | 6/426 (1.4) | 6/201 (3) | 4/165 (2.4) | 0.390 |
| Autoimmune/autoinflammatory disease | 30/788 (3.8) | 16/426 (3.8) | 11/198 (5.6) | 3/164 (1.8) | 0.182 |
| **Medications, n/N (%)** | | | | | |
| ACE-I or ARB | 374 (44.8) | 222 (50.6) | 88 (40.5) | 64 (35.5) | 0.003 |
| Ca antagonists | 259 (31) | 143 (32.6) | 67 (30.9) | 49 (27.2) | 0.569 |
| Beta blockers | 304 (36.4) | 161 (36.7) | 84 (38.7) | 59 (32.8) | 0.743 |
| Other antihypertensives | 19.4 (22.9) | 88 (20.1) | 39 (18) | 35 (19.4) | 0.798 |
| Insulin | 172 (20.6) | 84 (19.2) | 48/187 (25.7) | 40/152 (26.3) | 0.488 |
| Oral antidiabetics | 189/718 (26.3) | 103/379 (27.2) | 46/186 (24.7) | 40/153 (26.1) | 0.824 |
| Statins | 144/707 (20.4) | 71/371 (19.1) | 36/183 (19.7) | 37/153 (24.2) | 0.412 |
| Antiaggregant or anticoagulant drugs | 379/741 (51.1) | 196/386 (50.8) | 103/196 (52.6) | 80/159 (50.3) | 0.896 |
| **Smoking status, n/N (%)** | | | | | 0.477 |
| Never | 302/569 (53.1) | 173/323 (53.6) | 79/141 (56) | 50/105 (47.6) |  |
| Current | 50/569 (8.8) | 31/323 (9.6) | 8/141 (5.7) | 11/105 (10.5) |  |
| Former | 217/569 (38.1) | 119/323 (36.8) | 54/141 (38.3) | 44/105 (41.9) |  |
| **COVID-19 RT-PCR test** | | | | | 0.646 |
| Negative | 182/760 (23.9) | 101/413 (24.5) | 48/191 (25.1) | 33/156 (21.2) |  |
| Positive | 578/760 (76.1) | 312/413 (75.5) | 143/191 (74.9) | 123/156 (78.8) |  |
| **Symptoms related to COVID-19, n/N (%)** | | | | | |
| Fever | 509/811 (62.8) | 270/436 (61.9) | 135/204 (66.2) | 104/171 (60.8) | 0.491 |
| Fatigue | 497/803 (61.9) | 264/428 (61.7) | 136/206 (66) | 97/169 (57.4) | 0.229 |
| Dyspnea | 552/818 (67.5) | 268/435 (61.6) | 149/209 (71.3) | 135/174 (77.6) | <0.001 |
| Cough | 561/808 (69.4) | 300/432 (69.4) | 141/206 (68.4) | 120/170 (70.6) | 0.904 |
| Anorexia | 169/732 (23.1) | 106/407 (26) | 36/180 (20) | 27/145 (18.6) | 0.100 |
| Myalgia | 262/766 (34.2) | 146/419 (34.8) | 66/190 (34.7) | 50/157 (31.8) | 0.784 |
| Headache | 126/766 (16.4) | 69/414 (16.7) | 34/192 (17.7) | 23/160 (14.4) | 0.692 |
| Sore throat | 90/769 (11.7) | 50/420 (11.9) | 26/191 (13.6) | 14/158 (8.9) | 0.382 |
| Diarrhea | 50/779 (6.4) | 22/425 (5.2) | 16/194 (8.2) | 12/160 (7.5) | 0.289 |
| Others | 49/722 (6.8) | 28/395 (7.1) | 11/177 (6.2) | 10/150 (6.7) | 0.927 |
| Asymptomatic | 6/733 (0.8) | 5/396 (1.3) | 1/183 (0.5) | 0/154 (0) | 0.371 |
| **Possible source of COVID-19, n/N (%)** | | | | | 0.527 |
| Family-house | 153/741 (20.6) | 88/393 (22.4) | 37/195 (19) | 28/153 (18.3) |  |
| Nursing home or prison | 12/741 (1.6) | 6/393 (1.5) | 3/195 (1.5) | 3/153 (2) |  |
| Health institution | 25/741 (3.4) | 10/393 (2.5) | 9/195 (4.6) | 6/153 (3.9) |  |
| Social life (meeting or dinner) | 97/741 (13.1) | 43/393 (10.9) | 37/195 (19) | 17/153 (11.1) |  |
| Travel abroad | 14/741 (1.9) | 3/393 (0.8) | 7/195 (3.6) | 4/153 (2.6) |  |
| Domestic travel | 2/741 (0.3) | 1/393 (0.3) | 1/195 (0.5) | 0/153 (0) |  |
| Unknown | 438/741 (59.1) | 242/393 (61.6) | 101/195 (51.8) | 95/153 (62.1) |  |
| **Radiologic examination** | | | | | |
| Patients with a chest CT scan | 818/828 (98.8) | 429/434 (98.8) | 214/217 (98.6) | 175/177 (98.9) | 1.000 |
| Patients with specific chest CT findings | 779/817 (95.3) | 406/430 (94.4) | 204/212 (96.2) | 169/175 (96.6) | 0.407 |
| Patients with specific bilaterally chest CT findings | 686/797 (86.1) | 356/421 (84.6) | 182/210 (86.7) | 148/166 (89.2) | 0.336 |
| **Specific chest CT findings, n/N (%)** | | | | | |
| Ground glass opacity | 740/782 (94.6) | 390/412 (94.7) | 196/208 (94.2) | 154/162 (95.1) | 0.939 |
| Reticular opacity | 238/619 (38.4) | 123/319 (38.6) | 61/169 (36.1) | 54/131 (41.2) | 0.663 |
| Bronchial wall thickening | 129/617 (20.9) | 70/317 (22.1) | 35/169 (20.7) | 24/131 (18.3) | 0.671 |
| Pleural effusion | 168/623 (27) | 65/318 (20.4) | 54/171 (31.6) | 49/134 (36.6) | 0.001 |
| Thoracic lymphadenopathy | 99/613 (16.2) | 48/316 (15.2) | 28/166 (16.9) | 23/131 (17.6) | 0.791 |
| **Oxygen saturation in room air at diagnosis, n/N (%)** | | | | | <0.001 |
| Normal | 168/769 (21.8) | 112/407 (27.5) | 37/200 (18.5) | 19/162 (11.7) |  |
| 90-95% | 306/769 (39.8) | 185/407 (45.5) | 71/200 (35.5) | 50/162 (30.9) |  |
| <90% | 295/769 (38.4) | 110/407 (27) | 92/200 (46) | 93/162 (57.4) |  |
| **Severity of COVID-19 infection, n/N (%)** | | | | | <0.001 |
| Asymptomatic | 17/835 (2) | 12/438 (2.7) | 4/217 (1.8) | 1/180 (0.6) |  |
| Mild | 267/835 (32) | 178/438 (40.6) | 64/217 (29.5) | 25/180 (13.9) |  |
| Moderate | 362/835 (43.4) | 185/438 (42.2) | 92/217 (42.4) | 85/180 (47.2) |  |
| Severe and life-threatening | 189/835 (22.6) | 63/438 (14.4) | 57/217 (26.3) | 69/180 (38.3) |  |
| **Time between first symptom and COVID-19 diagnosis (days)** | 3 (2-5) | 3 (2-5) | 3 (2-5) | 3 (2-5) | 0.527 |
| **Data for renal function within the last year** | | | | | |
| Serum creatinine (µmol/L) | 88.4 (70.7-114.9) | 88.4 (70.7-114.9) | 79.6 (88.4-106.1) | 88.4 (70.7-132.6) | 0.049 |
| eGFR level (ml/min/1.73m^2^) | 64.1 (38.9-93.5) | 61.9 (38.2-91.2) | 66.6 (41.4-97.8) | 64.5 (31.2-94.7) | 0.236 |
| **CKD stages in patients with known eGFR**  **within the last year, n/N (%)** | | | | | 0.192 |
| eGFR>90 ml/min/1.73m2 | 175/656 (26.7) | 88/347 (25.4) | 49/172 (28.5) | 38/137 (27.7) |  |
| eGFR 60-90 ml/min/.73m^2^ | 181/656(27.6) | 97/347 (28.0) | 49/172 (28.5) | 35/137(25.5) |  |
| CKD stage 3 | 186/656 (28.4) | 103/347 (29.7) | 48/172 (27.9) | 35/137 (25.5) |  |
| CKD stage 4 | 82/656(12.3) | 49/347 (13.3) | 20/172 (11.6) | 15/137 (10.9) |  |
| CKD stage 5 | 33/656 (5) | 13/347 (3.7) | 6/172 (3.5) | 14/137 (10.2) |  |
| **Laboratory parameters at hospital admission** | | | | | |
| Urea (mmol/L) | 9.6 (6.3-15.0) | 9.1 (6.0-13.0) | 10.0 (6.6-16.2) | 12.1 (7.1-21.6) | <0.001 |
| Creatinine (µmol/L) | 123.8 (88.4-176.8) | 120.7 (91.3-147.6) | 123.8 (87.5-176.8) | 147.6 (84.4-291.7) | <0.001 |
| Na (mmol/L) | 137 (133-140) | 137 (134-140) | 136 (133-140) | 136 (132-140) | 0.194 |
| K (mmol/L) | 4.4 (4-4.9) | 4.4 (4-4.9) | 4.335 (3.9-4.9) | 4.4 (4-5) | 0.428 |
| AST (U/L) | 32 (20-50) | 28 (19-43) | 35 (21-58) | 38 (22-60) | <0.001 |
| ALT (U/L) | 22 (14-35) | 22 (14-33) | 22 (15-36) | 23 (14-46) | 0.203 |
| LDH (U/L) | 317 (240-445) | 294.5 (229-411) | 320 (240-468) | 380 (288-536) | <0.001 |
| Albumin (g/L) | 34.0 (29.5-38.0) | 36.0 (31.4-39.0) | 32.0 (27.1-37.0) | 31.0 (28.0-36.0) | <0.001 |
| Ferritin (µg/L) | 331 (152-737) | 271 (137-629) | 370.5 (151-876.5) | 471.5 (276-930.5) | <0.001 |
| Fibrinogen (g/L) | 4.7 (3.5-6.2) | 4.6 (3.3-60.1) | 4.6 (3.5-5.9) | 5.1 (4.1-6.6) | 0.009 |
| D-dimer (mg/L) | 14.8 (8.4-28.4) | 12.9 (7.3-24.0) | 16.9 (9.2-34.9) | 16.0 (10.8-32.8) | 0.006 |
| Procalcitonin (ng/L) | 380 (120-1200) | 220 (100-830) | 550 (130-1990) | 840 (225-1860) | <0.001 |
| Hemoglobin (g/dl) | 11.9 (10.2-13.7) | 12.5 (10.7-14) | 11.6 (10-13.3) | 11.1 (9.7-12.7) | <0.001 |
| Leucocyte count(/mm3) | 8395 (5790-12120) | 8100 (5600-11810) | 8255 (5790-12195) | 9800 (6140-13700) | 0.065 |
| Neutrophil count (/mm3) | 6100 (3840-9800) | 5760 (3670-9000) | 6035 (3950-9850) | 7540 (4500-10820) | 0.003 |
| Lymphocyte count (/mm3) | 1130 (740-1600) | 1245 (855-1700) | 1100 (730-1460) | 900 (600-1500) | <0.001 |
| Thrombocyte count (x1000/mm3) | 210 (152-285) | 219 (163-294) | 200 (141-279) | 200 (144-268) | 0.011 |
| CRP levels^†^**,** n/N (%) | | | | | <0.001 |
| Normal | 39/833 (4.7) | 29/438 (6.6) | 6/216 (2.8) | 4/179 (2.2) |  |
| 1/5-fold x ULN | 141/833 (16.9) | 95/438 (21.7) | 31/216 (14.4) | 15/179 (8.4) |  |
| 5/10-fold x ULN | 144/833 (17.3) | 76/438 (17.4) | 39/216 (18.1) | 29/179 (16.2) |  |
| 10/20-fold x ULN | 191/833 (22.9) | 105/438 (24) | 44/216 (20.4) | 42/179 (23.5) |  |
| >20-fold x ULN | 318/833 (38.2) | 133/438 (30.4) | 96/216 (44.4) | 89/179 (49.7) |  |
| **Specific treatments for COVID-19, n/N (%)** | | | | | |
| Hydroxychloroquine | 794/835 (95.1) | 421/438 (96.1) | 201/217 (92.6) | 172/180 (95.6) | 0.143 |
| Oseltamivir | 427/835 (51.1) | 244/438 (55.7) | 105/217 (48.4) | 78/180 (43.3) | 0.013 |
| Macrolide | 668/835 (80) | 365/438 (83.3) | 175/217 (80.6) | 128/180 (71.1) | 0.002 |
| Favipiravir | 473/835 (56.6) | 226/438 (51.6) | 127/217 (58.5) | 120/180 (66.7) | 0.002 |
| Glucocorticoid | 167/835 (20) | 54/438 (12.3) | 47/217 (21.7) | 66/180 (36.7) | <0.001 |
| Lopinavir-ritonavir | 48/835 (5.7) | 19/438 (4.3) | 14/217 (6.5) | 15/180 (8.3) | 0.134 |
| Tocilizumab | 79/835 (9.5) | 45/438 (10.3) | 14/217 (6.5) | 20/180 (11.1) | 0.201 |
| Convalescent plasma | 23/835 (2.8) | 9/438 (2.1) | 4/217 (1.8) | 10/180 (5.6) | 0.034 |
| Apheresis/immunoadsorption | 10/835 (1.2) | 2/438 (0.5) | 1/217 (0.5) | 7/180 (3.9) | 0.002 |
| IL-1 inhibitors (anakinra/canakinumab) | 2/835 (0.2) | 1/438 (0.2) | 0/217 (0) | 1/180 (0.6) |  |
| JAK2 inhibitors | 1/835 (0.1) | 1/438 (0.2) | 0/217 (0) | 0/180 (0) |  |
| **Unfavorable prognostic signs at any time during hospitalization, n/N (%)** | | | | | |
| Lymphopenia | 614/834 (73.6) | 303/437 (69.3) | 169/217 (77.9) | 142/180 (78.9) | 0.013 |
| Anemia (Hb <10 g/dL) | 446/834 (53.5) | 183/437 (41.9) | 125/217 (57.6) | 138/180 (76.7) | <0.001 |
| Thrombocytopenia | 261/830 (31.4) | 119/437 (27.2) | 71/215 (33) | 71/178 (39.9) | 0.008 |
| LDH (>2-fold x ULN) ^‡^ | 400/811 (49.3) | 173/431 (40.1) | 109/209 (52.2) | 118/171 (69) | <0.001 |
| AST (>2-fold x ULN) ^‡‡^ | 350/831 (42.1) | 138/436 (31.7) | 103/217 (47.5) | 109/178 (61.2) | <0.001 |
| Macrophage activation syndrome | 165/751 (22) | 60/401 (15) | 47/193 (24.4) | 58/157 (36.9) | <0.001 |
| Shock/severe hypotension | 289/789 (36.6) | 79/417 (18.9) | 91/203 (44.8) | 119/169 (70.4) | <0.001 |
| Secondary bacterial infection | 336/760 (44.2) | 122/401 (30.4) | 99/193 (51.3) | 115/166 (69.3) | <0.001 |
| CRP levels^†^**,** n/N (%) | | | | | <0.001 |
| Normal | 20/835 (2.4) | 19/438 (4.3) | 1/217 (0.5) | 0/180 (0) |  |
| 1/5-fold x ULN | 70/835 (8.4) | 50/438 (11.4) | 14/217 (6.5) | 6/180 (3.3) |  |
| 5/10-fold x ULN | 91/835 (10.9) | 60/438 (13.7) | 22/217 (10.1) | 9/180 (5) |  |
| 10/20-fold x ULN | 161/835 (19.3) | 96/438 (21.9) | 41/217 (18.9) | 24/180 (13.3) |  |
| >20-fold x ULN | 493/835 (59) | 213/438 (48.6) | 139/217 (64.1) | 141/180 (78.3) |  |
| **Intensive care unit admission, n/N (%)** | 428/835 (51.3) | 151/438 (34.5) | 126/217 (58.1) | 151/180 (83.9) | <0.001 |
| **Processes in the intensive care unit, n/N (%)** | | | | | |
| Intubation | 332/424 (78.3) | 95/151 (62.9) | 101/124 (81.5) | 136/149 (91.3) | <0.001 |
| ECMO | 19/363 (5.2) | 5/135 (3.7) | 2/97 (2.1) | 12/131 (9.2) | <0.001 |
| Slow continuous dialysis | 65/359 (18.1) | 14/131 (10.7) | 12/98 (12.2) | 39/130 (30) | <0.001 |
| **Duration of stay in intensive care unit (days)** | 9 (5-16) | 8.5 (4-18) | 8 (5-16) | 9 (6-16) | 0.851 |
| **AKI timing, n/N (%)** | | | | | |
| AKI at hospital admission | 404/835 (48.4) | 2196/438 (50.0) | 93/217 (42.9) | 92/180 (51.1) | 0.161 |
| AKI during hospital stay | 431/835 (51.6) | 219/438 (52.1) | 124/217 (57.1) | 88/180 (48.9) | 0.161 |
| **Suspected causes of AKI, n/N (%)** | | | | | <0.001 |
| Prerenal | 368/835 (44.1) | 255/438 (58.2) | 81/217 (37.3) | 32/180 (17.8) |  |
| Renal | 441/835 (52.8) | 171/438 (39) | 132/217 (60.8) | 138/180 (76.7) |  |
| Postrenal | 10/835 (1.2) | 4/438 (0.9) | 1/217 (0.5) | 5/180 (2.8) |  |
| Others | 16/835 (1.9) | 8/438 (1.8) | 3/217 (1.4) | 5/180 (2.8) |  |
| **Suspected specific causes of AKI, n/N (%)** |  | | | | <0.001 |
| Dehydration | 202/835 (24.2) | 150/438 (34.2) | 37/217 (17.1) | 15/180 (8.3) |  |
| GIS loss | 14/835 (1.7) | 9/438 (2.1) | 3/217 (1.4) | 2/180 (1.1) |  |
| Heart failure | 26/835 (3.1) | 12/438 (2.7) | 11/217 (5.1) | 3/180 (1.7) |  |
| Other prerenal causes | 126/835 (15.1) | 84/438 (19.2) | 30/217 (13.8) | 12/180 (6.7) |  |
| Sepsis | 318/835 (38.1) | 108/438 (24.7) | 94/217 (43.3) | 116/180 (64.4) |  |
| Thrombotic microangiopathy | 16/835 (1.9) | 7/438 (1.6) | 3/217 (1.4) | 6/180 (3.3) |  |
| Extended prerenal causes | 56/835 (6.7) | 28/438 (6.4) | 20/217 (9.2) | 8/180 (4.4) |  |
| Rhabdomyolysis | 4/835 (0.5) | 1/438 (0.2) | 1/217 (0.5) | 2/180 (1.1) |  |
| Nephrotoxic drugs | 47/835 (5.6) | 27/438 (6.2) | 14/217 (6.5) | 6/180 (3.3) |  |
| Postrenal (urological) causes | 10/835 (1.2) | 4/438 (0.9) | 1/217 (0.5) | 5/180 (2.8) |  |
| Others | 16/835 (1.9) | 8/438 (1.8) | 3/217 (1.4) | 5/180 (2.8) |  |
| **Time between hospitalization and AKI diagnosis (days)** | 4 (3-8) | 4 (2-6) | 5 (3-8) | 5 (3-9) | 0.031 |
| **Dialysis requirement in the ward, n/N (%)** | 133/799 (16.6) | 18/429 (4.2) | 24/199 (12.1) | 91/171 (53.2) | <0.001 |
| **KRT indications, n/N (%)** | | | | | |
| Increase in serum BUN/creatinine levels | 60/158 (38) | 10/26 (38.5) | 11/33 (33.3) | 39/99 (39.4) |  |
| Hyperkalemia | 17/158 (10.8) | 2/26 (7.7) | 4/33 (12.1) | 11/99 (11.1) |  |
| Metabolic acidosis | 34/158 (21.5) | 5/26 (19.2) | 7/33 (21.2) | 22/99 (22.2) |  |
| Hypervolemia | 31/158 (19.6) | 4/26 (15.4) | 6/33 (18.2) | 21/99 (21.2) |  |
| Severe uremic symptoms | 3/158 (1.9) | 1/26 (3.8) | 0/33 (0) | 2/99 (2) |  |
| Others | 13/158 (8.2) | 4/26 (15.4) | 5/33 (15.2) | 4/99 (4) |  |
| **Renal Outcome, n/N (%)** | | | | | <0.001 |
| Complete recovery | 402/835 (48.1) | 294/438 (67.1) | 87/217 (40.1) | 21/180 (11.7) |  |
| Partial recovery | 109/835 (13.1) | 56/438 (12.8) | 33/217 (15.2) | 20/180 (11.1) |  |
| No improvement and/or dialysis dependence | 21/835 (2.5) | 3/438 (0.7) | 3/217 (1.4) | 15/180 (8.3) |  |
| Undetermined | 303/835 (36.3) | 85/438 (19.4) | 94/217 (43.3) | 124/180 (68.9) |  |
| **Duration of AKI in discharged patients (days)** | 6 (3-9) | 5 (3-8) | 6 (4-10) | 8 (5-14) | <0.001 |
| **Total hospital stays (days)** | 12 (8-18) | 11 (7-17) | 12 (8-18) | 13.5 (9-21) | 0.007 |
| **Patient Outcome, n/N (%)** | | | | | <0.001 |
| Discharged | 515/835 (61.7) | 353/438 (80.6) | 116/217 (53.5) | 46/180 (25.6) |  |
| Dead | 320/835 (38.3) | 85/438 (19.4) | 101/217 (46.5) | 134/180 (74.4) |  |

ACE-I, angiotensin-converting enzyme inhibitors; ARB, angiotensin receptor blockers; COVID-19, coronavirus disease 2019; eGFR, estimated glomerular filtration rate; AST, aspartate aminotransferase; ALT, alanine aminotransferase; LDH, lactate dehydrogenase; x ULN, increase above upper normal limit; CRP, C-reactive protein; IL-1, interleukin 1; JAK2, Janus kinase; ECMO, extracorporeal membrane oxygenation; KRT, kidney replacement therapy

Data were expressed as median [Q1-Q3] or as number (percent)

^†^The upper limit of the normal range of CRP was 5 mg/L (47.6 nmol/L)

^‡^The upper limit of the normal range of LDH was 248 U/L

^‡‡^The upper limit of the normal range of AST was 37 U/L
