# Supplementary material for: Characteristics and outcomes of acute kidney injury in hospitalized COVID-19 patients: A multicenter study by the Turkish society of nephrology
Source: PLoS One. 2021 Aug 10;16(8):e0256023. doi: 10.1371/journal.pone.0256023 (PMC8354466; doi:10.1371/journal.pone.0256023)
Supplement: S2 Table — (DOCX) [file pone.0256023.s002.docx]

**S2 Table. Characteristics of COVID-19 RT-PCR positive patients, by the timing of initial development of AKI**

| **Variable** |  | **AKI timing** | |  | |  |
| --- | --- | --- | --- | --- | --- | --- |
|  | **Total (n=578)** | **At hospital admission (n=251)** | **During hospital stay (n=327)** | **P** | | |
| **Age (years)** | 69 (59-77.2) | 69 (59-78) | 69 (59-77) | 0.440 | | |
| **Male, n/N (%)** | 352/578 (60.9) | 157/251 (62.5) | 195/327 (59.6) | 0.452 | | |
| **Comorbid conditions, n/N (%)** | | | | | | |
| Diabetes mellitus | 249/569 (43.8) | 108/245 (44.1) | 141/324 (43.5) | 0.932 | | |
| Hypertension | 399/566 (70.5) | 187/243 (77) | 212/323 (65.6) | 0.004 | | |
| Chronic kidney disease | 192/510 (37.6) | 100/225 (44.4) | 92/285 (32.3) | 0.006 | | |
| Obesity | 27/462 (5.8) | 12/214 (5.6) | 15/248 (6.0) | 1.000 | | |
| Chronic obstructive pulmonary disease | 89/349 (16.2) | 37/238 (15.5) | 52/311 (16.7) | 0.728 | | |
| Coronary heart disease | 172/541 (31.8) | 81/236 (34.3) | 91/305 (29.8) | 0.309 | | |
| Heart failure | 85/528 (16.1) | 43/234 (18.4) | 42/294 (14.3) | 0.233 | | |
| Cerebrovascular disease | 39/546 (7.1) | 16/238 (6.7) | 23/308 (7.5) | 0.867 | | |
| Cancer | 69/554 (12.5) | 25/236 (10.6) | 44/318 (13.8) | 0.298 | | |
| Chronic liver disease | 10/556 (1.8) | 5/238 (2.1) | 5/318 (5.6) | 0.751 | | |
| Autoimmune/autoinflammatory disease | 25/556 (4.5) | 9/239 (3.8) | 16/317 (5.0) | 0.539 | | |
| **Medications, n/N (%)** | | | | | | |
| ACE-I or ARB | 266/505 (52.7) | 132/227 (58.1) | 134/278 (48.2) | 0.031 | | |
| Ca antagonists | 171/491 (34.8) | 71/224 (31.7) | 100/267 (37.5) | 0.181 | | |
| Beta blockers | 216/491 (44.0) | 105/223 (47.1) | 111/268 (41.4) | 0.235 | | |
| Other antihypertensives | 113/476 (23.7) | 41/214 (19.2) | 72/262 (27.5) | 0.040 | | |
| Insulin | 122/484 (25.2) | 61/225 (27.1) | 61/259 (40.8) | 0.402 | | |
| Oral antidiabetics | 127/486 (26.1) | 55/223 (24.7) | 72/263 (27.4) | 0.535 | | |
| Statins | 88/477 (18.4) | 42/221 (19.0) | 46/256 (18.0) | 0.813 | | |
| Antiaggregant or anticoagulant drugs | 251/501 (50.1) | 110/228 (48.2) | 141/273 (51.6) | 0.473 | | |
| **Smoking status, n/N (%)** 0.319 | | | | | | |
| Never | 221/411 (53.8) | 100/200 (50.0) | 121/211 (57.3) |  | | |
| current | 33/411 (8.0) | 18/200 (9.0) | 15/211 (7.1) |  | | |
| former | 157/411 (38.2) | 82/200 (41.0) | 75/211 (35.5) |  | | |
| **Oxygen saturation in room air at diagnosis, n/N (%)** 0.569 | | | | | | |
| Normal | 129/551 (23.4) | 61/239 (25.5) | 68/312 (21.8) |  | | |
| 90-95% | 210/551 (38.1) | 90/239 (37.7) | 120/312 (38.5) |  | | |
| <90% | 212/551 (38.5) | 88/239 (36.8) | 123/312 (39.7) |  | | |
| **Time between first symptom and COVID-19 diagnosis (days)** | 3 (2-5) | 3 (2-4) | 2 (2.2-5) | 0.168 | | |
| **Severity of COVID-19 infection, n/N (%)** 0.324 | | | | | | |
| Asymptomatic | 13/578 (2.2) | 4/251 (1.6) | 9/327 (2.8) |  | | |
| Mild to moderate | 196/578 (33.9) | 78/251 (31.1) | 118/327 (36.1) |  | | |
| Severe | 256/578 (44.3) | 121/251 (48.2) | 135/327 (41.3) |  | | |
| Critical | 113/578 (19.6) | 48/251 (19.1) | 65/327 (19.9) |  | | |
| **Data of renal function within the last year.** | | | | | | |
| Serum creatinine (µmol/L) | 86.6 (70.7-109.6) | 88.4 (77.4-114.1) | 80.5 (68.8-113.2) | 0.005 | | |
| eGFR (mL/min/1.73 m^2^) | 65.4 (40.6-96.6) | 60.9 (38.7-88.2) | 70.7 (44.6-106.5) | 0.012 | | |
| **Laboratory parameters at hospital admission** | | | | | | |
| Urea (mmol/L) | 9.3 (6.0-14.0) | 12.5 (9.1-18.6) | 7.0 (5.2-10.0) | <0.001 | | |
| Creatinine (µmol/L) | 117.6 (88.4-164.5) | 150.3 (127.3-214.0) | 92.0 (76.0-115.8) | <0.001 | | |
| Na (mmol/L) | 137 (134-140) | 136 (133-140) | 137 (134-140) | 0.182 | | |
| K (mmol/L) | 4.4 (3.9-4.8) | 4.5 (4.0-5.0) | 4.2 (3.8-4.6) | <0.001 | | |
| AST (U/L) | 32 (20-49.4) | 33 (20.0-52.5) | 31 (20-47) | 0.168 | | |
| ALT (U/L) | 23 (14-35) | 24 (14-40) | 22 (14-34) | 0.117 | | |
| LDH (U/L) | 320 (239-443) | 328.5 (247-452) | 314 (229.5-437) | 0.141 | | |
| Albumin (g/L) | 34.7 (30-38.9) | 34.6 (30.7-38) | 35 (30-39) | 0.516 | | |
| Ferritin (µg/L) | 260 (160.5-750) | 407 (166.2-788) | 311 (157.6-742.5) | 0.310 | | |
| Fibrinogen (g/L) | 4.7 (3.5-6.2) | 4.6 (3.4-6.2) | 4.8 (3.6-6.2) | 0.602 | | |
| D-dimer (mg/L) | 15.1 (8.1-27.9) | 13.8 (7.8-28.1) | 14.6 (9.3-27.1) | 0.761 | | |
| Procalcitonin (ng/L) | 350 (130-1147.5) | 320 (120-1190) | 380 (130-1130) | 0.889 | | |
| Hemoglobin (g/dl) | 12.1 (10.6-13.8) | 11.9 (11-14.2) | 12.2 (10.8-13.8) | 0.415 | | |
| Leucocyte count (/mm3) | 7835 (5500-11262) | 7650 (5600-11810) | 8000 (5485-10097.5) | 0.697 | | |
| Neutrophil count (/mm3) | 5750 (3700-9110) | 5600 (3785-9205) | 6500 (4190-9230) | 0.967 | | |
| Lymphocyte count (/mm3) | 1100 (700-1520) | 1170 (800-1585) | 1035 (662.5-1500) | 0.037 | | |
| Thrombocyte count (x1000/mm3) | 202.5 (151-274.5) | 210 (160-284) | 195 (144.5-260.5) | 0.024 | | |
| CRP levels^†^, n/N (%) |  |  |  | 0.049 | | |
| Normal | 28/576 (5.5) | 7/251 (2.8) | 21/325 (6.5) |  | | |
| 1/5-fold x ULN | 83/576 (16.3) | 37/251 (14.7) | 54/325 (16.6) |  | | |
| 5/10-fold x ULN | 94/576 (18.5) | 40/15.9 (15.9) | 63/325 (19.4) |  | | |
| 10/20-fold x ULN | 136/576 (26.7) | 78/251 (31.1) | 72/325 (22.2) |  | | |
| >20-fold x ULN | 168/576 (33.0) | 89/251 (35.5) | 115/325 (35.4) |  | | |
| **Unfavorable prognostic signs at any time**  **during hospital stay, n/N (%)** | | | | | | |
| Lymphopenia | 443/578 (76.6) | 181/251 (72.1) | 262/327 (80.1) | 0.029 | | |
| Anemia (Hb <10 g/dL) | 293/578 (50.7) | 120/251 (47.8) | 173/327 (50.7) | 0.240 | | |
| Thrombocytopenia | 189/574 (32.9) | 74/249 (29.7) | 115/325 (34.5) | 0.179 | | |
| LDH (>2-fold x ULN) ^‡^ | 303/559 (54.2) | 126/241 (52.3) | 177/318 (55.7) | 0.441 | | |
| AST (>2-fold x ULN) ^‡‡^ | 254/575 (44.2) | 103/249 (41.4) | 151/326 (46.3) | 0.271 | | |
| Macrophage activation syndrome | 124/519 (23.9) | 42/229 (18.3) | 82/290 (28.3) | 0.009 | | |
| Shock/severe hypotension | 213/557 (38.2) | 77/245 (31.4) | 136/312 (43.6) | 0.004 | | |
| Secondary bacterial infection | 247/531 (46.5) | 103/235 (43.8) | 144/296 (48.6) | 0.294 | | |
| CRP levels^†^**,** n/N (%) |  |  |  | 0.289 | | |
| Normal | 17/578 (2.9) | 5/251 (2.0) | 12/327 (3.7) |  |  |  |
| 1/5-fold x ULN | 40/578 (6.9) | 22/251 (8.8) | 18/327 (5.5) |  |  |  |
| 5/10-fold x ULN | 59/578 (10.2) | 28/251 (11.2) | 31/327 (9.5) |  |  |  |
| 10/20-fold x ULN | 124/578 (21.5) | 57/251 (22.7) | 67/327 (20.5) |  |  |  |
| >20-fold x ULN | 238/578 (58.5) | 139/251 (55.4) | 199/327 (60.9) |  |  |  |
| **Intensive care unit admission, n/N (%)** | 291/578 (50.3) | 107/251 (42.6) | 184/327 (56.3) | 0.001 | | |
| **Managements in the intensive care unit , n/N (%)** | | | | | | |
| Intubation | 232/288 (80.6) | 80/106 (75.5) | 152/182 (83.5) | 0.122 | | |
| ECMO | 18/265 (6.8) | 7/97 (7.2) | 11/168 (6.5) | 0.806 | | |
| Slow continuous dialysis | 53/260 (20.4) | 23/94 (24.5) | 30/166 (18.1) | 0.262 | | |
| **Duration of stay in intensive care unit (days)** | 9 (5.8-17) | 8 (5-16) | 10 (6-18) | 0.109 | | |
| **Suspected causes of AKI, n/N (%)** 0.103 | | | | | | |
| Prerenal | 251/578 (43.4) | 116/251 (46.2) | 135/327 (41.3) |  | | |
| Renal | 311/578 (53.8) | 125/251 (49.8) | 186/327 (56.9) |  | | |
| Postrenal | 6/578 (1.0) | 5/251 (2.0) | 1/327 (0.3) |  | | |
| Others | 10/578 (1.7) | 5/251 (2.0) | 5/327 (1.5) |  | | |
| **Suspected specific causes of AKI, n/N (%)** 0.125 | | | | | | |
| Dehydration | 141/578 (24.4) | 69/251 (27.5) | 72/327 (22.0) |  | | |
| GIS loss | 10/578 (1,7) | 4/251 (1.6) | 6/327 (1,8) |  | | |
| Heart failure | 19/578 (3.3) | 10/251 (4.0) | 9/327 (2.8) |  | | |
| Other prerenal causes | 81/578 (14.0) | 33/251 (13.1) | 58/327 (14.7) |  | | |
| Sepsis | 233/578 (40.3) | 147/251 (45.0) | 157/327 (45.0) |  | | |
| Thrombotic microangiopathy | 8/578 (1.4) | 5/251 (2.0) | 3/327 (0.9) |  | | |
| Extended prerenal causes | 36/578 (6,2) | 18/251 (7.2) | 18/327 (5.5) |  | | |
| Rhabdomyolysis | 2/578 (0,3) | 2/251 (0,8) | 0/327 (0) |  | | |
| Nephrotoxic drugs | 32/578 (5,5) | 14/251 (5.6) | 18/327 (5.5) |  | | |
| Postrenal (urological) causes | 6/578 (1.0) | 5/251 (2.0) | 1/327 (0.3) |  | | |
| Others | 10/578 (1,7) | 5/251 (2.0) | 5/327 (1.5) |  | | |
| **AKI Stage, n/N (%)** 0.170 | | | | | | |
| Stage 1 | 312/578 (54.0) | 145/251 (57.8) | 167/327 (51.1) |  | | |
| Stage 2 | 143/578 (24.7) | 53/251 (21.1) | 90/327 (27.5) |  | | |
| Stage 3 | 123/578 (21.3) | 53/251 (21.1) | 70/327 (21.4) |  | | |
| **Dialysis requirement in the ward, n/N (%)** | 91/554 (16.4) | 37/236 (15.7) | 54/318 (17.0) | 0.729 | | |
| **KRT indications, n/N (%)**  0.716 | | | | | | |
| Increase in serum BUN/creatinine levels | 42/115 (36.5) | 15/47 (31.9) | 27/68 (39.7) |  | | |
| Hyperkalemia | 10/115 (8.7) | 6/47 (12.8) | 4/68 (5.9) |  | | |
| Metabolic acidosis | 27/115 (23.5) | 11/47 (23.4) | 16/68 (23.5) |  | | |
| Hypervolemia | 22/115 (19.1) | 9/47 (19.1) | 13/68 (19.1) |  | | |
| Severe uremic symptoms | 3/115 (2.6) | 2/47 (4.3) | 1/68 (1.5) |  | | |
| Others | 11/115 (9.6) | 4/47 (8.5) | 7/68 (10.3) |  | | |
| **Renal Outcome, n/N (%)** |  |  |  | 0.900 | | |
| Complete recovery | 285/349 (81.7) | 138/171 (80.7) | 147/178 (82.6) |  | | |
| Partially recovery | 60/349 (17.2) | 31/171 (18.1) | 29/178 (16.3) |  | | |
| Dialysis dependence | 4/349 (1.1) | 2/171 (1.2) | 2/178 (1.1) |  | | |
| **Duration of AKI in discharged patients (days)** | 6 (3-9) | 6 (4-10) | 5 (3-8) | 0.001 | | |
| **Patient Outcome, n/N (%)** 0.003 | | | | | | |
| Discharged | 353/578 (61.1) | 171/251 (68.1) | 182/327 (55.7) |  | | |
| Dead | 225/578 (38.9) | 80/251 (31.9) | 145/327 (44.3) |  | | |
| **Total hospital stays (days)** | 12 (8-19) | 12 (8-16) | 13 (9-21) | 0.002 | | |

ACE-I, angiotensin-converting enzyme inhibitors; ARB, angiotensin receptor blockers; COVID-19, coronavirus disease 2019; eGFR, estimated glomerular filtration rate; AST, aspartate aminotransferase; ALT, alanine aminotransferase; LDH, lactate dehydrogenase; x ULN, increase above upper normal limit; CRP, C-reactive protein; IL-1, interleukin 1; JAK2, Janus kinase; ECMO, extracorporeal membrane oxygenation; KRT, kidney replacement therapy

Data were expressed as median [Q1-Q3] or as number (percent)

^†^The upper limit of the normal range of CRP was 5 mg/L (47.6 nmol/L)

^‡^The upper limit of the normal range of LDH was 248 U/L

^‡‡^The upper limit of the normal range of AST was 37 U/L
