# Supplementary material for: Characteristics and outcomes of acute kidney injury in hospitalized COVID-19 patients: A multicenter study by the Turkish society of nephrology
Source: PLoS One. 2021 Aug 10;16(8):e0256023. doi: 10.1371/journal.pone.0256023 (PMC8354466; doi:10.1371/journal.pone.0256023)
Supplement: S3 Table — (DOCX) [file pone.0256023.s003.docx]

**S3 Table. Predictors associated with acute kidney injury developed hospital stay by Cox regression analysis**

| **Variables** | **Cox regression analysis** | | |
| --- | --- | --- | --- |
|  | HR | 95% CI | p |
| Chronic kidney disease | 1,160 | 0.891-1.512 | 0.270 |
| Hypertension | 0.965 | 0.740-1.258 | 0.792 |
| Intensive care admission | 1.586 | 1.238-2.032 | <0.001 |
| Lymphopenia during hospitalization | 1.473 | 1.238-2,032 | 0.010 |
