# Supplementary material for: Characteristics and outcomes of acute kidney injury in hospitalized COVID-19 patients: A multicenter study by the Turkish society of nephrology
Source: PLoS One. 2021 Aug 10;16(8):e0256023. doi: 10.1371/journal.pone.0256023 (PMC8354466; doi:10.1371/journal.pone.0256023)
Supplement: S4 Table — (DOCX) [file pone.0256023.s004.docx]

**S4 Table. Some characteristics of COVID-19 RT-PCR positive patients, by CKD status**

| **Variable** |  | **Baseline Chronic Kidney Disease** | | |
| --- | --- | --- | --- | --- |
|  | **Total (n=510)** | **No (n=318)** | **Yes (n=192)** | **P** |
| **Possible source of COVID-19, n/N (%)** 0.701 | | | | |
| Family-house | 127/486 (26.1) | 75/301 (24.9) | 52/185 (28.1) |  |
| Health institution | 20/486 (4.1) | 13/301 (4.3) | 7/185 (3.8) |  |
| Social life (meeting or dinner) | 57/486 (11.7) | 39/301 (13.0) | 18/185 (9.7) |  |
| Travel abroad | 10/486 (2.1) | 6/301 (2.0) | 4/185 (2.2) |  |
| Domestic travel | 1/486 (0.2) | 0/301 (0.0) | 1/285 (0.5) |  |
| Unknown | 261/486 (53.7) | 163/301 (54.2) | 98/185 (53.0) |  |
| **Radiologic examination, n/N (%)** | | | | |
| Patients with a chest CT scan | 495/505 (98.0) | 306/314 (97.5) | 189/191 (99.0) | 0.332 |
| Patients with specific chest CT findings | 461/488 (94.5) | 280/300 (93.3) | 181/188 (96.3) | 0.499 |
| Patients with specific bilaterally chest CT findings | 418/488 (85.7) | 252/300 (84.0) | 166/188 (83.3) | 0.232 |
| **Specific chest CT findings, n/N (%)** | | | | |
| Ground glass opacity | 445/475 (93.5) | 268/292 (91.8) | 176/183 (96.2) | 0.084 |
| Reticular opacity | 141/377 (37.4) | 79/242 (32.6) | 62/135 (45.9) | 0.014 |
| Bronchial wall thickening | 74/377 (19.6) | 40/240 (16.7) | 34/137 (24.8) | 0.060 |
| Pleural effusion | 91/375 (24.3) | 48/236 (20.3) | 43/139 (30.9) | 0.025 |
| Thoracic lymphadenopathy | 49/373 (13.1) | 23/238 (9.7) | 26/135 (19.3) | 0.011 |
| **Time between first symptom and COVID-19 diagnosis (days)** | 3.0 (3.3-4.1) | 3.0 (3.2-4.5) | 3.0 (2.9-4.2) | 0.021 |
| **Specific treatments for COVID-19, n/N (%)** | | | | |
| Hydroxychloroquine | 417/510 (97.5) | 308/318 (96.9) | 189/192 (98.4) | 0.388 |
| Oseltamivir | 282/519 (55.3) | 183/318 (57.5) | 99/192 (51.6) | 0.199 |
| Macrolide | 418/510 (82.0) | 263/318 (82.7) | 155/192 (80.7) | 0.635 |
| Favipiravir | 315/510 (61.8) | 201/318 (63.2) | 114/192 (59.4) | 0.399 |
| Glucocorticoid | 82/510 (16.1) | 57/318 (17.9) | 25/192 (13.0) | 0.171 |
| Lopinavir-ritonavir | 30/510 (5.9) | 26/318 (8.2) | 4/192 (2.1) | 0.003 |
| Tocilizumab | 62/510 (12.2) | 47/318 (14.8) | 15/192 (7.8) | 0.025 |
| Convalescent plasma | 15/510 (2.9) | 12/318 (3.8) | 3/192 (1.6) | 0.184 |
| Apheresis/immunoadsorption | 8/510 (1.6) | 6/318 (1.9) | 2/192 (1.0) | 0.716 |
| JAK2 inhibitors | 0/510 (0.0) | 0/318 (0.0) | 0/192 (0.0) |  |

COVID-19, coronavirus disease 2019; JAK2, Janus kinase

Data were expressed as number (percent)
