# Supplementary material for: Characteristics and outcomes of acute kidney injury in hospitalized COVID-19 patients: A multicenter study by the Turkish society of nephrology
Source: PLoS One. 2021 Aug 10;16(8):e0256023. doi: 10.1371/journal.pone.0256023 (PMC8354466; doi:10.1371/journal.pone.0256023)
Supplement: S5 Table — (DOCX) [file pone.0256023.s005.docx]

**S5 Table. Some characteristics of COVID-19 RT-PCR positive patients, by patient survival**

| **Variable** | **Total**  **(n=578)** | **Discharged (n=353)** | **Dead (n=225)** | **p** |
| --- | --- | --- | --- | --- |
| **Possible source of COVID-19, n/N (%)** |  |  |  |  |
| Family-house | 139/541 (25.7) | 99/331 (29.9) | 40/210 (19) |  |
| Nursing home or prison | 10/541 (1.8) | 6/331 (1.8) | 4/210 (1.9) |  |
| Health institution | 21/541 (3.9) | 9/331 (2.7) | 12/210 (5.7) |  |
| Social life (meeting or dinner) | 68/541 (12.6) | 44/331 (13.3) | 24/210 (11.4) |  |
| Travel abroad | 10/541 (1.8) | 5/331 (1.5) | 5/210 (2.4) |  |
| Domestic travel | 1/541 (0.2) | 1/331 (0.3) | 0/210 (0) |  |
| Unknown | 292/541 (54) | 167/331 (50.5) | 125/210 (59.5) |  |
| **Specific treatments for COVID-19, n/N (%)** | | | | |
| Hydroxychloroquine | 560/578 (96.9) | 344/353 (97.5) | 216/225 (96) | 0.328 |
| Oseltamivir | 324/578 (56.1) | 180/353 (51) | 144/225 (64) | 0.002 |
| Macrolide | 471/578 (81.5) | 294/353 (83.3) | 177/225 (78.7) | 0.163 |
| Favipiravir | 369/578 (63.8) | 197/353 (55.8) | 172/225 (76.4) | <0.001 |
| Glucocorticoid | 111/578 (19.2) | 28/353 (7.9) | 83/225 (36.9) | <0.001 |
| Lopinavir-ritonavir | 39/578 (6.7) | 16/353 (4.5) | 23/225 (10.2) | 0.008 |
| Tocilizumab | 73/578 (12.6) | 34/353 (9.6) | 39/225 (17.3) | 0.007 |
| Convalescent plasma | 19/578 (3.3) | 2/353 (0.6) | 17/225 (7.6) | <0.001 |
| Apheresis/immunoadsorption | 9/578 (1.6) | 1/353 (0.3) | 8/225 (3.6) | 0.003 |
| JAK2 inhibitors | 1/578 (0.2) | 0/353 (0) | 1/225 (0.4) |  |
| **Suspected specific causes of AKI, n/N (%)** |  |  |  |  |
| Dehydration | 141/578 (24.4) | 132/353 (37.4) | 9/225 (4) |  |
| GIS loss | 10/578 (1.7) | 10/353 (2.8) | 0/225 (0) |  |
| Heart failure | 19/578 (3.3) | 12/353 (3.4) | 7/225 (3.1) |  |
| Other prerenal causes | 81/578 (14) | 66/353 (18.7) | 15/225 (6.7) |  |
| Sepsis | 233/578 (40.3) | 69/353 (19.5) | 164/225 (72.9) |  |
| Thrombotic microangiopathy | 8/578 (1.4) | 3/353 (0.8) | 5/225 (2.2) |  |
| Extended prerenal causes | 36/578 (6.2) | 23/353 (6.5) | 13/225 (5.8) |  |
| Rhabdomyolysis | 2/578 (0.3) | 1/353 (0.3) | 1/225 (0.4) |  |
| Nephrotoxic drugs | 32/578 (5.5) | 28/353 (7.9) | 4/225 (1.8) |  |
| Postrenal (urological) causes | 6/578 (1) | 5/353 (1.4) | 1/225 (0.4) |  |
| Others | 10/578 (1.7) | 4/353 (1.1) | 6/225 (2.7) |  |
| **Dialysis indications, n/N (%)** | | | | |
| İncrease in serum BUN/creatinine levels | 42/115 (36.5) | 6/15 (40) | 36/100 (36) |  |
| Hyperkalemia | 10/115 (8.7) | 0/15 (0) | 10/100 (10) |  |
| Metabolic acidosis | 27/115 (23.5) | 1/15 (6.7) | 26/100 (26) |  |
| Hypervolemia | 22/115 (19.1) | 5/15 (33.3) | 17/100 (17) |  |
| Severe uremic symptoms | 3/115 (2.6) | 0/15 (0) | 3/100 (3) |  |
| Others | 11/115 (9.6) | 3/15 (20) | 8/100 (8) |  |

COVID-19, coronavirus disease 2019

Data were expressed as number (percent)
