# Supplementary material for: Characteristics and outcomes of acute kidney injury in hospitalized COVID-19 patients: A multicenter study by the Turkish society of nephrology
Source: PLoS One. 2021 Aug 10;16(8):e0256023. doi: 10.1371/journal.pone.0256023 (PMC8354466; doi:10.1371/journal.pone.0256023)
Supplement: S6 Table — (DOCX) [file pone.0256023.s006.docx]

**S6 Table. Characteristics of COVID-19 positive RT-PCR patients according to renal outcomes**

| **Variable** | **Total** | **Complete recovery (n=285)** | **Partially recovery (n=60)** | **No improvement in renal function (n=1) or dialysis-dependent (n=3)** |  |
| --- | --- | --- | --- | --- | --- |
| Age (years) | 67 (57-77) | 67 (55-76) | 71.5 (60-77.7) | 66.5 (57.2-78) | 0.313 |
| Male, n/N (%) | 200/349 (57.3) | 163/285 (57.2) | 33/60 (55) | 4/4 (100) | 0.211 |
| **Comorbid conditions, n/N (%)** | | | | | |
| Diabetes mellitus | 143/345 (41.4) | 120/282 (42.6) | 21/60 (35) | 2/3 (66.7) | 0.376 |
| Hypertension | 252/342 (73.7) | 205/279 (73.5) | 44/60 (73.3) | 3/3 (100) | 0.582 |
| Chronic kidney disease | 124/333 (37.2) | 93/285 (32.6) | 29/46 (63) | 2/2 (100) | <0.001 |
| Obesity | 18/289 (6.2) | 16/235 (6.8) | 2/52 (11.1) | 0/2 (0) | 0.679 |
| Chronic obstructive lung disease | 53/339 (15.6) | 44/277 (15.9) | 9/59 (15.3) | 0/3 (0) | 0.750 |
| Coronary heart disease | 98/334 (29.3) | 79/271 (29.2) | 18/60 (30) | 1/3 (33.3) | 0.980 |
| Heart failure | 50/327 (15.3) | 37/266 (13.9) | 12/58 (20.7) | 1/3 (33.3) | 0.192 |
| Cerebrovascular disease | 14/337 (4.2) | 13/277 (4.7) | 1/57 (1.8) | 0/3 (0) | 0.561 |
| Cancer | 24/338 (7.1) | 20/276 (7.2) | 4/59 (6.8) | 0/3 (0) | 0.884 |
| Chronic liver disease | 5/339 (1.5) | 5/276 (1.8) | 0/60 (0) | 0/3 (0) | 0.560 |
| **Severity of COVID-19 infection, n/N (%)** |  |  |  |  | 0.039 |
| Asymptomatic | 12/349 (3.4) | 12/285 (4.2) | 0/60 (0) | 0/4 (0) |  |
| Mild to moderate | 169/349 (46.7) | 143/285 (50.2) | 23/60 (38.3) | 0/4 (0) |  |
| Severe | 145/349 (41.5) | 108/285 (37.9) | 33/60 (55) | 4/4 (100) |  |
| Critical | 27/349 (7.4) | 22/285 (7.7) | 4/60 (6.7) | 0/4 (0) |  |
| **Serum creatinine (µmol/L)** **within the last year** | 82.4 (71.6-120.2) | 82.4 (70.7-108.7) | 104.3 (79.6-162.7) | 223.7 (194.5-) | 0.010 |
| **CKD stages in patients with known eGFR**  **within the last year, n/N (%)** |  |  |  |  | 0.002 |
| eGFR>90 ml/min/1.73m2 | 66/270 (24.4) | 58/222 (26.1) | 8/46 (17.4) | 0/2 (0) |  |
| eGFR 60-90 ml/min/.73m^2^ | 80/270 (29.6) | 71/222 (32.0) | 9/46 (19.6) | 0/2 (0) |  |
| CKD stage 3 | 68/270 (25.2) | 58/222 (26.1) | 10/46 (21.7) | 0/2 (0) |  |
| CKD stage 4 | 39/270 (14.4) | 25/222 (11.3) | 13/46 (28.3) | 1/2(50) |  |
| CKD stage 5 | 17/270 (6.3) | 10/222 (4.5) | 6/46 (13) | 1/2 (50) |  |
| **Laboratory parameters at hospital admission** | | | | | |
| Urea (mmol/L) | 9.3 (6.0-13.6) | 8.8 (5.7-12.5) | 11.2 (8.1-17.8) | 112.6 (18.7-27.5) | <0.001 |
| Creatinine (µmol/L) | 123.8 (94.6-161.8) | 120.2 (93.7-150.3) | 154.7 (101.5-247.6) | 538.5 (324.5-706.5) | <0.001 |
| Na (mmol/L) | 137 (134-140) | 137 (134-140) | 136 (133-140) | 136.5 (135.2-138.5) | 0.662 |
| K (mmol/L) | 4.4 (4-4.8) | 4.4 (4-4.8) | 4.6 (4.2-5.0) | 4.8 (4.6-5.3) | 0.016 |
| AST (U/L) | 26.5 (19-40) | 26 (18-39.6) | 33 (20.2-46) | 20.2 (17.7-41.9) | 0.116 |
| ALT (U/L) | 21 (14-32.5) | 21 (13-32) | 23 (14-34.7) | 12.5 (10.8-22.7) | 0.334 |
| LDH (U/L) | 285 (223.5-385) | 280 (228.5-384) | 289 (210-420) | 358.5 (314.7-655.7) | 0.915 |
| Albumin (g/L) | 37 (32.6-39.8) | 37 (33-40) | 35.8 (31.8-39) | 35.3 (32.3-38.7) | 0,054 |
| Ferritin (µg/L) | 267 (137-632) | 272 (137.2-631.5) | 211 (126-545) | 457 (79.5-1658.7) | 0.207 |
| Fibrinogen (g/L) | 4.5 (3.3-6.0) | 4.6 (3.5-6.3) | 4.0 (2.8-5.0) | 4.0 (2.9-5.5) | 0.004 |
| D-dimer (mg/L) | 13.5 (7.9-24.0) | 13.1 (6.7-24.0) | 13.3 (10-22) | 22.0 (10.9-) | 0.548 |
| Procalcitonin (ng/L) | 230 (100-790) | 200 (97.5-770) | 270 (130-760) | 128 (18.2-2456) | 0.094 |
| Hemoglobin (g/dl) | 12.4 (11-14) | 12.6 (11.1-14) | 12.1 (10.7-13.8) | 10.1 (9.1-11) | 0.048 |
| Leucocyte count(/mm3) | 7420 (5335-10210) | 7460 (5300-10173.5) | 7390 (5530-11595) | 7950 (6335-11230) | 0.169 |
| Neutrophil count (/mm3) | 5300 (3600-7810) | 5300 (3500-7810) | 5270.5 (3607-7707.5) | 5750 (4647.5-8105) | 0.472 |
| Lymphocyte count (/mm3) | 1200 (900-1600) | 1200 (887.5-1600) | 1225 (930-1622) | 1300 (610-1675) | 0.856 |
| Thrombocyte count (x1000/mm3) | 209.5 (160.6-280) | 209.5 (160.6-280) | 214 (162.5-292.7) | 175.8 (139.4-217.7) | 0.345 |
| CRP levels^†^**,** n/N (%) |  |  |  |  | 0.576 |
| Normal | 25/348 (7.2) | 23/284 (8.1) | 2/60 (3.3) | 0/4 (0) |  |
| 1/5-fold x ULN | 75/348 (21.6) | 60/284 (21.1) | 14/60 (23.3) | 1/4 (25) |  |
| 5/10-fold x ULN | 72/348 (20.7) | 58/284 (20.4) | 14/60 (23.3) | 0/4 (0) |  |
| 10/20-fold x ULN | 89/348 (25.6) | 76/284 (26.8) | 11/60 (18.3) | 2/4 (50) |  |
| >20-fold x ULN | 89/348 (25.0) | 67/284 (23.6) | 19/60 (31.7) | 1/4 (25) |  |
| **Unfavorable prognostic signs at any time during hospital stay , n/N (%)** |  |  |  |  |  |
| Lymphopenia | 249/347 (70.8) | 203/285 (71.2) | 40/60 (66.7) | 4/4 (100) | 0.338 |
| Anemia (Hb <10 g/dL) | 134/349 (38.4) | 101/285 (35.4) | 29/60 (48.3) | 4/4 (100) | 0.007 |
| Thrombocytopenia | 81/349 (23.2) | 65/285 (22.8) | 15/60 (25) | 1/4 (25) | 0.932 |
| LDH (>2-fold x ULN) ^‡^ | 139/343 (40.5) | 109/279 (39.1) | 28/60 (46.7) | 2/4 (50) | 0.513 |
| AST (>2-fold x ULN) ^‡‡^ | 101/349 (28.9) | 86/285 (30.2) | 15/60 (25) | 0/4 (0) | 0.318 |
| Macrophage activation syndrome | 32/336 (9.5) | 28/273 (10.3) | 3/59 (5.1) | 1/4 (25) | 0.268 |
| Shock/severe hypotension | 21/338 (6.2) | 17/274 (6.2) | 4/60 (6.7) | 0/4 (0) | 0.867 |
| Secondary bacterial infection | 94/337 (27.9) | 75/276 (27.2) | 17/57 (29.8) | 2/4 (50) | 0.563 |
| CRP levels^†^ |  |  |  |  | 0.194 |
| Normal | 17/349 (4.9) | 17/285 (6.0) | 0/60 (0) | 0/4 (0) |  |
| 1/5-fold x ULN | 36/349 (10.3) | 30/285 (10.5) | 6/60 (10) | 0/4 (0) |  |
| 5/10-fold x ULN | 51/349 (14.6) | 38/285 (13.3) | 13/60 (21.7) | 0/4 (0) |  |
| 10/20-fold x ULN | 90/349 (25.8) | 74/285 (26.0) | 16/60 (26.7) | 0/4 (0) |  |
| >20-fold x ULN | 155/349 (44.4) | 126/285 (44.2) | 25/60 (41.7) | 4/4 (100) |  |
| **Intensive care unit admission, n/N (%)** | 69/349 (19.8) | 59/285 (20.7) | 9/60 (15) | 1/4 (25) | 0.581 |
| **Managements in the intensive care unit, n/N (%)** | | | | | |
| Intubation | 25/340 (7.4) | 18/279 (6.5) | 6/58 (10.3) | 1/3 (33.3) | 0.131 |
| Slow continuous dialysis | 9/339 (2.7) | 8/278 (2.9) | 1/58 (1.7) | 0/3 (0) | 0.848 |
| Duration of stay in intensive care unit (days) | 8.5 (6-16.5) | 8 (5.2-14.7) | 18 (8.7-46.2) | 11 (11-11) | 0.003 |
| **AKI Stage, n/N (%)** |  |  |  |  | 0.001 |
| Stage 1 | 251/349 (71.9) | 213/285 (74.7) | 37/60 (61.7) | 1/4 (25) |  |
| Stage 2 | 76/349 (21.8) | 59/285 (20.7) | 16/60 (26.7) | 1/4 (25) |  |
| Stage 3 | 22/349 (6.3) | 13/285 (4.6) | 7/60 (11.7) | 2/4 (50) |  |
| **Suspected causes of AKI, n/N (%)** |  |  |  |  |  |
| Prerenal | 218/349 (62.5) | 183/285 (64.2) | 34/60 (56.7) | 1/4 (25) |  |
| Renal | 122/349 (35.0) | 94/285 (33.0) | 25/60 (41.7) | 3/4 (75) |  |
| Postrenal | 5/349 (1.4) | 4/285 (1.4) | 1/60 (1.7) | 0/4 (0) |  |
| Others | 4/349 (1.1) | 4/285 (1.4) | 0/60 (0) | 0/4 (0) |  |
| **Suspected specific causes of AKI, n/N (%)** |  |  |  |  |  |
| Dehydration | 131/349 (37.5) | 112/285 (39.3) | 18/60 (30) | 1/4 (25) |  |
| GIS loss | 10/349 (2.9) | 7/285 (2.5) | 3/60 (5) | 0/4 (0) |  |
| Heart failure | 12/349 (3.4) | 10/285 (3.5) | 2/60 (3.3) | 0/4 (0) |  |
| Other prerenal causes | 65/349 (18.6) | 54/285 (18.9) | 11/60 (18.3) | 0/4 (0) |  |
| Sepsis | 216/349 (40.8) | 56/285 (19.2) | 11/60 (18.3) | 1/4 (25) |  |
| Thrombotic microangiopathy | 3/349 (0.9) | 2/285 (0.7) | 1/60 (1.7) | 0/4 (0) |  |
| Extended prerenal causes | 22/349 (6.3) | 17/285 (6.0) | 4/60 (6.7) | 1/4 (25) |  |
| Rhabdomyolysis | 1/349 (0.3) | 0/285 (0) | 0/60 (0) | 1/4 (25) |  |
| Nephrotoxic drugs | 28/349 (8.0) | 19/285 (6.7) | 9/60 (15) | 0/4 (0) |  |
| Postrenal (urological) causes | 5/349 (1.4) | 4/285 (1.4) | 1/60 (1.7) | 0/4 (0) |  |
| Others | 4/349 (1.1) | 4/285 (1.4) | 0/60 (0) | 0/4 (0) |  |
| **AKI Stage, n/N (%)** |  |  |  |  |  |
| Stage 1 | 289/510 (56.7) | 186/318 (58.5) | 103/192 (53.6) |  |  |
| Stage 2 | 127/510 (24.9) | 80/318 (25.2) | 47192 (24.5) |  |  |
| Stage 3 | 94/510 (18.4) | 52/318 (16.4) | 42/192 (21.9) |  |  |
| **KRT requirement, n/N (%)** | 19/348 (5.5) | 15/284 (5.3) | 1/56 (1.7) | 3/4 (75) | <0.001 |
| **Total hospital stays (days)** | 12 (8-17) | 12 (8-17) | 12.5 (9.2-16) | 19 (11.5-22) | 0.091 |

COVID-19, coronavirus disease 2019; AST, aspartate aminotransferase; ALT, alanine aminotransferase; LDH, lactate dehydrogenase; x ULN, increase above upper normal limit; CRP, C-reactive protein; KRT, kidney replacement therapy

Data were expressed as median [Q1-Q3] or as number (percent)

^†^The upper limit of the normal range of CRP was 5 mg/L (47.6 nmol/L)

^‡^The upper limit of the normal range of LDH was 248 U/L

^‡‡^The upper limit of the normal range of AST was 37 U/L
